# From efficiency to resilience: unraveling the dynamic coupling of land use economic efficiency and urban ecological resilience in Yellow River Basin

**DOI:** 10.1038/s41598-024-67364-4

**Published:** 2024-07-17

**Authors:** Tonghui Yu, Shanshan Jia, Xufeng Cui

**Affiliations:** 1 School of Business, Xinyang Normal University, Xinyang, Henan People’s Republic of China; 2 School of Business Administration, Zhongnan University of Economics and Law, Wuhan, 430073 Hubei People’s Republic of China

**Keywords:** Yellow River Basin, Land use economic efficiency, Urban ecological resilience, Coupling coordination degree, Environmental economics, Environmental impact, Sustainability

## Abstract

This study investigates the Dynamic Coupling between Land Use Economic Efficiency (LUEE) and Urban Ecological Resilience (UER) in the Yellow River Basin (YRB). This exploration is pivotal for elucidating the interaction mechanisms between economic growth and ecological governance. Furthermore, understanding this relationship is essential for fostering high-quality, sustainable urban development in the YRB. Utilizing panel data from 56 cities spanning 2003 to 2020, this study employed the coupling coordination degree (CCD) model, spatial correlation analysis, Kernel density estimation, convergence model, and Geodetector to systematically analyze the spatio-temporal distribution, dynamic trend, and determinants of the CCD between LUEE and UER in the YRB. The findings indicate that: (1) A general upward trend in both LUEE and UER, accompanied by a steady improvement in their CCD. (2) Significant spatial disparities in their CCD, with higher levels in the lower reaches. (3) Marked positive spatial autocorrelation, predominantly characterized by clusters where high (low) values are surrounded by high (low) values. (4) Regarding the impact of individual factors, government fiscal budget expenditure demonstrates the most robust explanatory power for the CCD within the YRB. Concerning the effects of two-factor interactions, the interplay between industrial structure upgrading and government fiscal budget expenditure emerges as the most significant determinant in influencing the CCD between LUEE and UER. This study enhances our comprehensive understanding of the interplay between economic and ecological systems. It offers scientific insights and strategic direction for harmonizing ecological governance with urban economic growth at both the regional and global scales.

## Introduction

The acceleration of global urbanization has emerged as an undeniable phenomenon. From 1950 to 2021, the global urban population increased from 0.75 billion (World Bank, 1950) to 4.2 billion (United Nations, 2021). It is projected that by 2050, this number will rise to 6.4 billion, corresponding to an urbanization rate of 68%^1^. Urbanization, serving as a pivotal catalyst for enhancing the quality of economic growth, can facilitate high-quality economic development and the continual refinement of population size and composition. Nonetheless, the rapid pace of urbanization presents numerous challenges. Urbanization’s accelerated pace, largely relying on the extensive expansion of urban construction land, involves significant resource consumption and inevitably results in issues such as inefficient land utilization^2,3^, disorganized urban spatial configurations^4^, and the deterioration of natural ecosystems^5,6^. These challenges not only imperil the sustainable development of urban areas but also pose severe threats to the ecological environment. To effectively address these ecological issues, it is crucial to enhance ecosystem resilience while mitigating the conflict between urban economic growth and ecological preservation. Pursuing land use economic efficiency (LUEE) necessitates establishing and refining ecological compensation mechanisms to ensure sustainable land use and ecosystem stability^7,8^. Consequently, exploring the correlation between LUEE and urban ecological resilience (UER) is crucially important. This study offers a robust scientific foundation for optimizing urbanization quality and improving ecological conditions. It further aids in significantly enhancing urban sustainability and promotes the establishment of livable, green, and dynamic cities.

The pattern of urbanization in developing countries frequently exhibits “late start and rapid development”^9^. As the world’s largest developing nation, China confronts dual challenges of environmental pollution and resource scarcity while advancing urbanization^10^. Particularly, the Yellow River Basin (YRB), an area integrating fragile ecosystems and vital socio-economic elements, faces multiple issues such as severe soil erosion, increasing resource constraints, a marked decrease in biodiversity, and ecological deterioration^11,12^. Furthermore, data from the National Bureau of Statistics of China reveal that by 2021, the urbanization rate in the YRB had reached 61.3%, leading to population and economic concentration in key cities and urban agglomerations^13^. Nonetheless, this concentration is constrained by several factors, such as the carrying capacity of urban land, environment, and ecosystems. In response to these challenges, there is an urgent need to optimize the allocation of productive resources like land, reduce the negative externalities of environmental pollution, and enhance the resilience of ecosystems to adapt swiftly to new equilibrium states after disturbances, thereby ensuring the smooth functioning of basic living spaces and urban economic activities.

In this context, it is crucial to thoroughly explore the CCD between LUEE and UER during urbanization in the YRB. This involves revealing the coupling mechanisms and driving factors of these two systems. Such exploration is not only a practical necessity for regional coordinated development but also a fundamental requirement for scientifically implementing economic growth and ecological protection in the region. By promoting a rational urban planning layout, enhancing LUEE, reducing resource waste and environmental pollution, and strengthening the capacity of ecosystems to respond, adapt, and regenerate, we can significantly advance economic growth and ecological protection in the YRB^14^. This approach will also provide a more scientific basis for decision-making and offer theoretical support for the sustainable development of urbanization in developing countries, both in China and worldwide.

## Literature review

The rapid expansion of urban economies inevitably introduces increasing uncertainties and compounded risks. To effectively mitigate these challenges, urban ecological governance should pivot from merely reducing vulnerability to strengthening resilience^15^. Urban land, a vital component of ecological governance^16^, plays a crucial role in fostering economic growth and enhancing ecological environments. Over the past few years, there has been an escalating interest globally in the development of “resilient cities”, as observed by the international community. This movement aims to confront various unpredictable risk challenges, thereby bolstering the cities’ immunity and the ecosystems’ capacity for recovery^17^. The term “resilience” originates from the Latin “resilio”, meaning “to rebound”, was initially used in mechanics to denote the capacity of an object to withstand external forces and revert to its original state^18^. Holling (1973) was the pioneer in integrating the notion of resilience within the realm of natural ecology. He developed a theoretical framework for ecological resilience, underscoring the system’s capacity to regain stability after experiencing a disturbance^19^. The introduction of this concept has provided a vital theoretical reference for comprehending the steady-state balance and sustainable development of ecosystems. Gradually, its influence has extended across the field of ecological research^20^. Current research grounded in Holling’s (1973) theoretical framework primarily focuses on defining the concept of ecological resilience, constructing indicator systems, measuring resilience levels, and identifying influential factors. Additionally, it explores the interactions between ecological resilience and related variables^21,22^. Significantly, the interaction between ecological resilience and urbanization has emerged as a critical academic focus^23,24^. While exploring the coupling relationship between these two factors, it has been observed that urbanization establishes a connection with ecological resilience through various factors, including population growth, economic development, industrial structure upgrading, and spatial expansion^25,26^. Furthermore, as urbanization progresses rapidly, efficiently using land resources and preserving the ecology are now pivotal in attaining sustainable development in urban areas^27^. In this context, enhancing both LUEE and UER is regarded as a crucial approach to achieving sustainability. This approach not only facilitates economic growth but also minimizes negative environmental impacts.

A comprehensive review of the existing research shows that land use efficiency (LUE), a central concern in disciplines like geography and land management, has evolved through substantial theoretical and methodological advancements, establishing a research system characterized by rich content and diverse methods^28^. Existing literature predominantly emphasizes on the utilization efficiency across diverse land use categories, encompassing urban construction, agricultural, commercial, and residential lands. Comprehensive discussions are provided on their spatio-temporal patterns, driving mechanisms, spatial spillover effects, and interactions with other variables^29–31^. Additionally, current studies investigate the determinants of LUE across multiple dimensions. Factors such as economic development level, infrastructure construction, industrial structure upgrading, technological innovation, and policy support are identified as crucial drivers or constraints of LUE^32–35^. In contrast to LUE, the LUEE focuses more on the economic aspect, typically measured by the input–output ratio. Drawing from existing studies^36^, this paper employs inputs like labor, capital, and land, along with outputs like the value-added in secondary and tertiary industries, to evaluate LUEE. These indicators not only reflect regional socio-economic progress but also serve as crucial metrics for evaluating factor allocation and resource utilization efficiency^37,38^. In conclusion, an in-depth examination of LUEE is instrumental in optimizing land resource distribution, fostering balanced regional economic growth, and providing a theoretical foundation for sustainable, high-quality urban economic development.

The increasingly significant interactive linkages between economic and environmental systems have propelled scholars to pivot the research paradigm of urban economic development and ecological environmental protection from separate inquiries^39–41^ towards investigating their bidirectional coupling^42,43^. This shift facilitates a deeper understanding of the mutual impacts and mechanisms at play between the systems^44^. In recent years, the link between urban economic development and ecological environmental protection has become increasingly close. The expansion of urban construction land has supported economic and social development, and these activities in turn have placed higher demands on land use^27,36^. However, an overemphasis on economic growth may jeopardize ecosystems, leading to issues such as spatial expansion and fragile ecological functions^46^. Consequently, current research increasingly centers on the interaction between LUE and the ecological environment. These studies delve into the interrelationships, influence mechanisms, and evolution trends of land use concerning ecological efficiency, ecological carrying capacity, ecosystem services, carbon emission efficiency, etc.^1,47–49^, employing models such as gray correlation and coupling coordination degrees to quantify and unveil the interactive response dynamics between the economy and ecosystems^50,51^. By scrutinizing the nexus between the LUEE and UER, fresh theoretical underpinnings can be provided for optimizing urban land use patterns and fostering ecological civilization construction, thereby further advancing sustainable economic and social development.

**Figure 1:**
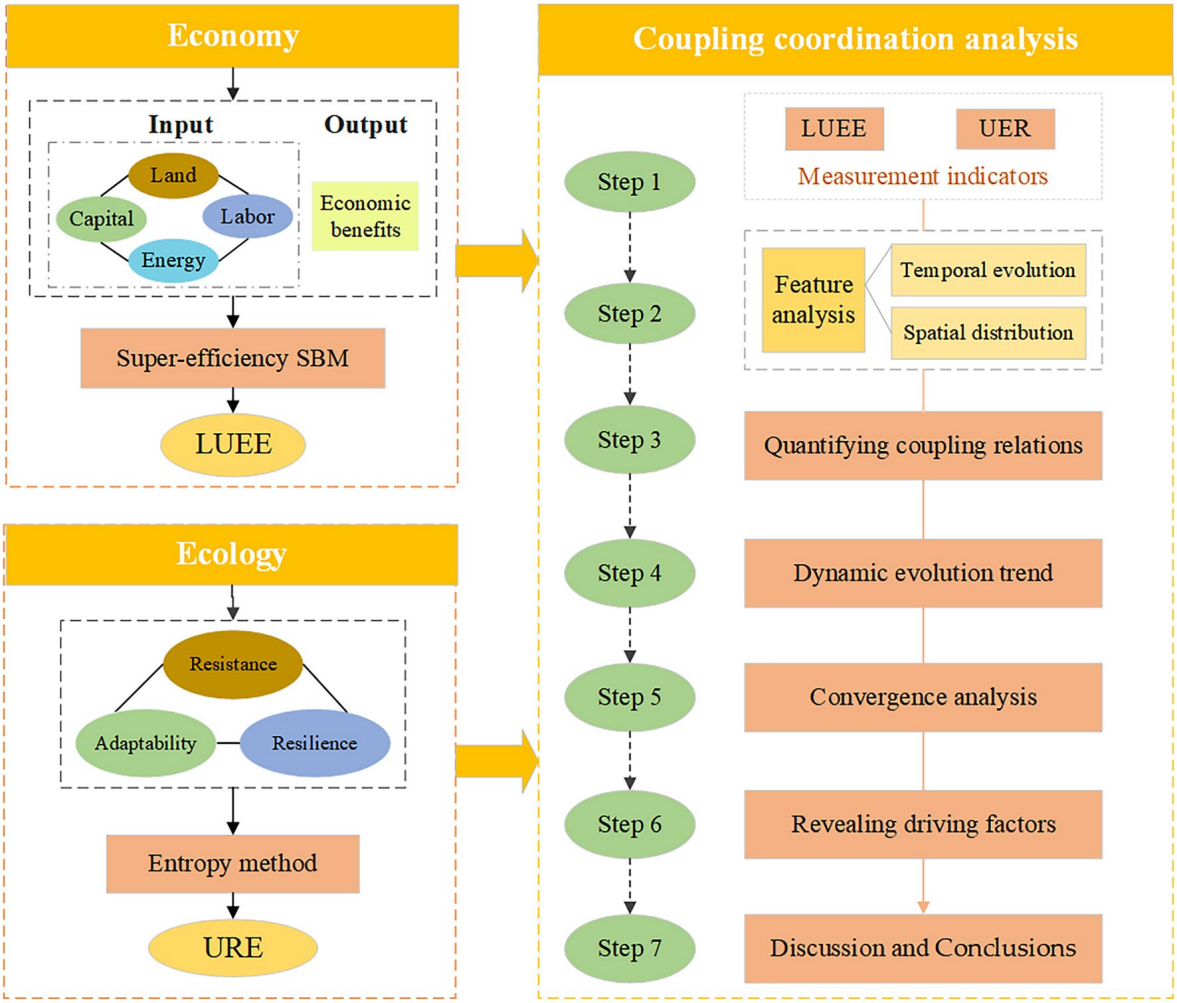
Research framework.

Taking an overview of existing studies, the research on LUE and UER has achieved certain results, laying a theoretical foundation for this study. Nevertheless, ample room remains for further investigation persist. Firstly, current research primarily concentrates on the measurement of LUE and the identification of its influencing factors, but lacks an in-depth exploration of LUEE, which is more emphasis on economic considerations. This gap may result in a one-sided understanding of LUEE, neglecting the actual impact of economic inputs. Secondly, research on the interaction between LUEE and UER is lacking, along with an effective understanding of synergistic optimization strategies between the two. This deficiency may hinder the full revelation of the complex interaction mechanisms between them, thereby affecting the comprehensive understanding of their interrelationship. Thirdly, the spatial and temporal evolution characteristics of the CCD between LUEE and UER, along with their driving forces, warrant deeper exploration. The lack of in-depth understanding of these features and drivers may prevent us from accurately grasping the dynamic changes between LUEE and UER, which in turn makes it difficult to formulate effective management and optimization strategies at different temporal and spatial scales. Additionally, the insufficient research on driving factors may hinder our ability to identify and leverage key elements to promote the CCD of LUEE and UER, potentially affecting the long-term sustainability of regional development. In light of these considerations, this study’s marginal contributions are as follows: firstly, building upon a comprehensive review of relevant literature, we underscore the economic significance of the LUEE indicator compared to the traditional LUE evaluation metric. Our approach focuses more on economic output in measurement methods, while considering energy inputs. Secondly, we systematically delineate the theoretical interaction mechanisms governing the coupling and coordination relationship between LUEE and UER from diverse perspectives. Through systematic analysis, we elucidate the interaction mechanism between them, thereby furnishing a theoretical foundation for understanding the synergistic relationship between economic development and ecological environmental protection in the urbanization process. Thirdly, we employ various research methodologies such as the super-efficient SBM model, the entropy method, and the CCD model to scientifically assess the level of coupling coordination between LUEE and UER in the YRB. Additionally, we identify key drivers affecting the CCD development of LUEE and UER (Fig. 1).

### Mechanism analysis

In recent years, the rapid urbanization process has led to the expansion of urban construction land, exacerbating pressure on the ecological environment while driving cities to generate economic benefits. Faced with increasingly severe resource and environmental challenges, enhancing the steady-state conversion power and self-organization capacity of cities becomes urgent and critical^42,52^. Therefore, we attempt to elucidate the coupling mechanism between LUEE and UER to promote urban economic development while ensuring the achievement of environmental sustainability goals.

LUEE can enhance ecological carrying capacity through internal technological progress, industrial structure upgrading, and policy orientation^1,53,61^. Firstly, regarding internal technological progress, improving economic efficiency can facilitate the dissemination of new green technologies, expand the external spillover effect of technology, and consequently enhance the pure technical efficiency and scale efficiency of land use, thereby alleviating pressure on the ecosystem. Secondly, concerning industrial structure upgrading, enhancing the LUEE utilization imposes higher demands on land use arrangements for industrial development, promoting optimal allocation of the industrial structure to achieve effective resource allocation and utilization and foster the synergistic development of the economy, society, and ecosystem. Thirdly, in terms of government policy orientation, the formulation and implementation of government policy in land use and economic development are crucial. This involves guiding rational development and utilization of land resources through scientific and reasonable land use planning, environmental protection laws and regulations, incentive policies, and strengthening government supervision to promote ecological protection and restoration.

**Figure 2:**
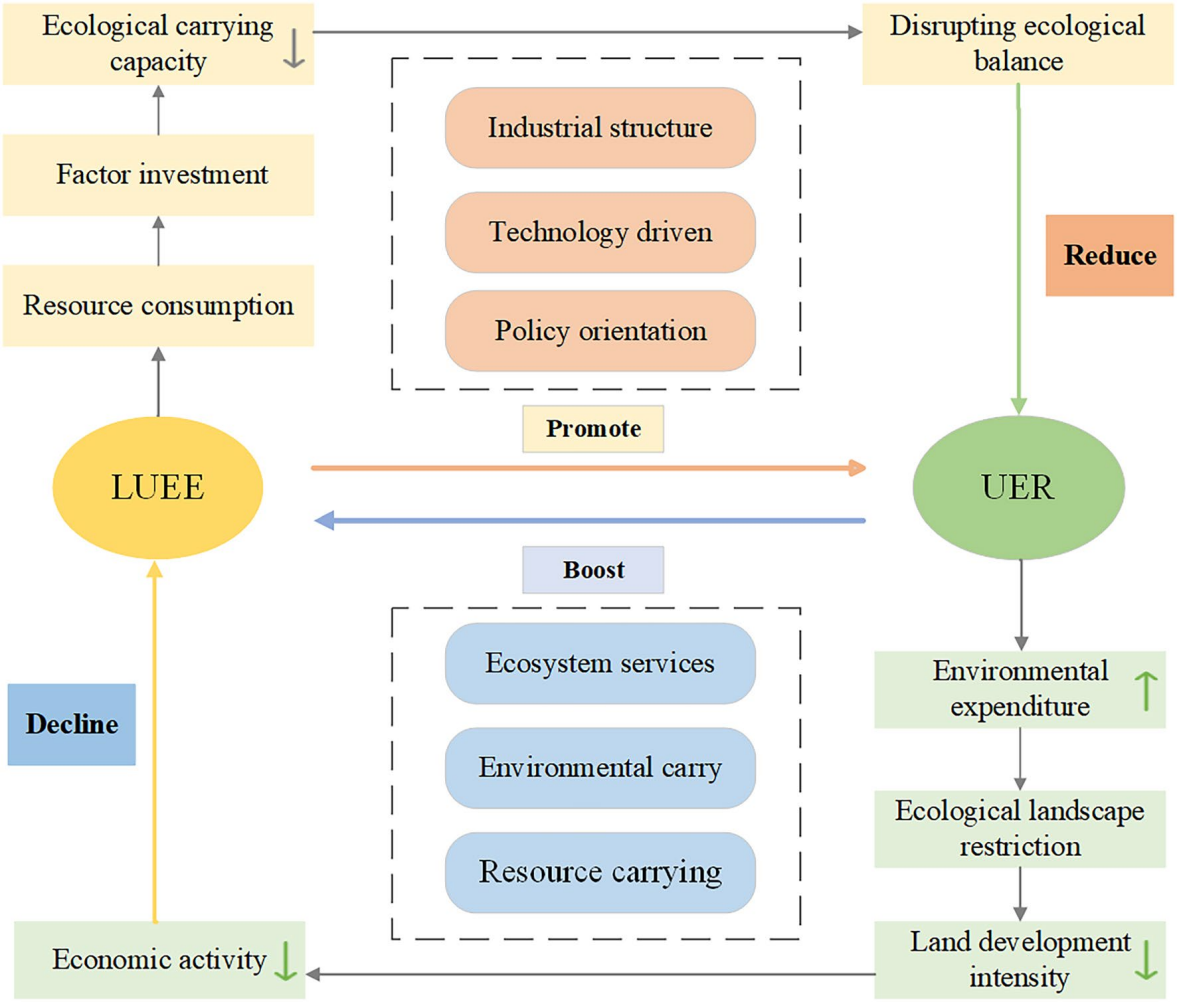
The Interactive Mechanism of LUEE and UER.

UER can achieve a stable improvement in the LUEE by increasing resource carrying capacity, enhancing environmental capacity, and optimizing ecosystem services. Firstly, concerning resource carrying capacity, enhancing it can improve the supply capacity of land resources, enabling cities to effectively absorb and utilize resources, reduce environmental loads, maintain urban ecological balance, and thus provide important support for improving the LUEE. Secondly, regarding environmental capacity, by improving the quality of the urban ecological environment and reducing environmental pollution, the city’s resilience to the external environment can be enhanced to ensure the long-term and stable enhancement of the LUEE^54,55^. Thirdly, concerning ecosystem services, optimizing ecosystem services not only creates a conducive environment for the city’s economic activities but also attracts more eco-tourism and recreational activities, thereby positively impacting the improvement of LUEE^8,74^. However, when the government increases financial investment in the ecological field, it may lead to the diversion of funds that could have been used for economic development, and excessive ecological protection may also lead to the underutilization of resources. For example, strict control over the development of natural landscapes such as mountains, lakes, and forests may limit the intensity of land development, which in turn affects the development of economic activities such as tourism and recreation, and to a certain extent reduces the LUEE (Fig. 2).

## Materials and methods

### Study area

**Figure 3:**
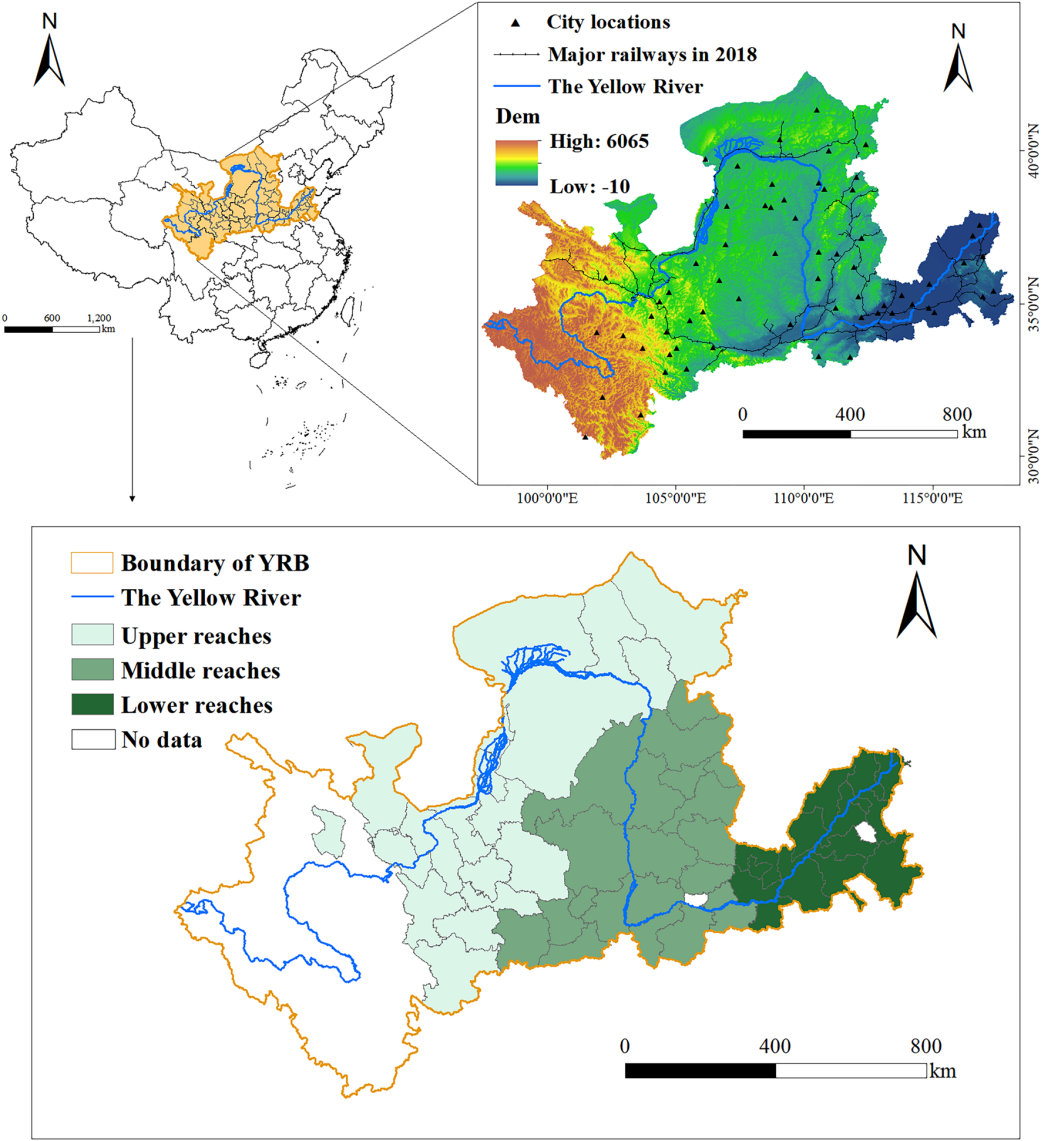
The study area map in the YRB.

The Yellow River, originating from the Bayan Har Mountains in Qinghai Province, is recognized as the world’s fifth-longest river and China’s second-largest. It traverses nine provinces, including Qinghai, Sichuan, Gansu, Ningxia, Inner Mongolia, Shaanxi, Shanxi, Henan, and Shandong, ultimately flowing into the Bohai Sea. Stretching approximately 5,464 km, its basin spans an expansive area of about 795,000 square kilometers (Fig. 3). Following *the State Council’s Guiding Opinions on Promoting the Yangtze River Economic Belt’s Development *via* the Golden Waterway*, Sichuan Province has been incorporated into the Yangtze River Economic Zone. Consequently, this study includes 56 cities across eight provinces within the YRB. Notably, Jiyuan City and Laiwu City are excluded due to significant data gaps during the research period. The categorization of cities into the upper, middle, and lower reaches of the YRB is derived from the standards in the *Yellow River Cultural Encyclopedia*^56^.

### Methodology

#### Super-efficiency SBM model

Data Envelopment Analysis (DEA) is a method employed for efficiency evaluation using various inputs and outputs and has been continually advancing with technological progress. Traditional DEA models, however, do not adequately address the slackness issues in input and output variables. Addressing this challenge, Tone (2001) introduced the SBM approach for directional distance measurement, integrating slack variables into the objective function. This innovation significantly enhances the model’s capacity to differentiate among various Decision Making Units (DMUs) and to track efficiency changes over time. In terms of comprehensively assessing the efficiency of a given system, the super-efficiency SBM model is able to comprehensively consider multiple input and output indicators, and compared with the traditional SBM model, it can further evaluate the decision-making units with an efficiency value greater than 1, thus providing more accurate efficiency assessment results. In addition, the super-efficient SBM model can flexibly handle multiple data types, including cross-sectional data, time series data, etc., and is applicable to the efficiency assessment of many fields, such as industry, agriculture, and service industry. Therefore, it has become an important efficiency assessment tool in academic research and practical applications. In light of these advancements, this study employs the super-efficient SBM model to quantify the LUEE. The formula for measurement is as follows:

1 $$\min \theta^{ * } = \frac{{1 + \frac{1}{m}\sum\limits_{m = 1}^{M} {\left( {S_{m}^{x} /S_{jm}^{t} } \right)} }}{{1 - \frac{1}{l + h}\sum\limits_{l = 1}^{L} {(S_{l}^{y} /y_{jl}^{t} } )}}$$

2 $$s.t.\left\{ {\begin{array}{*{20}l} {x_{jm}^{t} \ge \sum\limits_{j = 1,j \ne 0}^{n} {\lambda_{j}^{t} x_{jm}^{t} + S_{m}^{x} } } \hfill \\ \begin{gathered} \hfill \\ y_{jt}^{t} \ge \sum\limits_{j = 1,j \ne k}^{n} {\lambda_{j}^{t} y_{jt}^{t} + S_{l}^{y} } \hfill \\ \end{gathered} \hfill \\ {\lambda_{j}^{t} \ge 0,S_{m}^{x} \ge 0,S_{l}^{y} \ge 0,j = 1, \ldots n} \hfill \\ \end{array} } \right.$$

where θ* is the value of LUEE, $x_{jm}^{t}$ and $y_{jt}^{t}$ respectively represent the inputs and expected outputs of *DMU*_*j*_ in period *t*, $S_{m}^{x}$ and $S_{l}^{y}$ represent the slack variables of inputs and expected outputs, respectively; $\lambda$ is a vector of weights.

#### Entropy method

The entropy method is an objective analytical methodology based on data computation and remains unaffected by subjective biases. It establishes indicator weights by assessing the distribution and variability of indicator data, thereby minimizing the influence of subjective assessments and enhancing the objectivity of the evaluation outcomes. Consequently, the entropy method is utilized in this study to assess the UER of 56 cities within the YRB from 2003 to 2020. The process involves the following steps:

(1) Standardization of comprehensive evaluation indicators for UER:

3 $$X_{ij} = \frac{{x_{ij} - \min (x_{ij} )}}{{\max (x_{ij} ) - \min (x_{ij} )}}$$

4 $$X_{ij} = \frac{{\max (x_{ij} ) - x_{ij} }}{{\max (x_{ij} ) - \min (x_{ij} )}}$$

where *x*_*ij*_ represents the raw values of the *j* indicators of the evaluation unit *i*, *X*_*ij*_ is the result of the standardization of the comprehensive evaluation indicators.

(2) The weight of the *j*-*th* indicator in the *i*-*th* indicator:

5 $$p_{ij} = \frac{{X_{ij} }}{{\sum\nolimits_{i = 1}^{m} {X_{ij} } }}$$

(3) The information entropy *e*_*j*_:

6 $$e_{j} = - \frac{1}{\ln \left( m \right)}\sum\nolimits_{i = 1}^{m} {\left\{ {p_{ij} } \right.} \left. {\ln \left( {p_{ij} } \right)} \right\}$$

(4) The weight *w*_*j*_:

7 $$w_{j} = \frac{{1 - e_{j} }}{{\sum\nolimits_{j = 1}^{n} {\left( {1 - e_{j} } \right)} }}$$

(5) Each city’s *UER*_*i*_:

8 $$UER_{i} = \sum\nolimits_{j = 1}^{n} {w_{j} X_{ij} }$$

#### Coupling coordination degree (CCD) model

Coupling, characterized by the interaction and mutual influence of separate systems, leading to a synergistic effect, is quantifiable through CCD^57^. The CCD model is employed for examining the interactive synergy between LUEE and UER. The model’s formulation is as follows:

9 $$C = \left\{ {(U_{1} U_{2} )/\left( {\frac{{U_{1} + U_{2} }}{2}} \right)^{2} } \right\}^{\frac{1}{2}}$$

where *C* denotes the coupling degree, *U*_*1*_ is designated as the assessment of LUEE, *U*_*2*_ is the comprehensive measure of UER. To delve deeper into their coordination and compatibility, the model is extended to include the following formulation:

10 $$T = \alpha U_{1} + \beta U_{2}$$

11 $$D = \sqrt {CT}$$

where *T* signifies the overall coordination index for LUEE and UER. The weights for LUEE and UER, denoted by α and *β* respectively, are set equally at 0.5, reflecting their equal importance as per existing research^58,59^. *D* represents the CCD between LUEE and UER.

Utilizing the CCD values, the interrelation of LUEE and UER is classified into ten specific grades ^60,61^, as elaborated in Table 1.

**Table 1:** Grade division of the CCD between LUEE and UER.

Grade	D	Type	Grade	D	Type
I	0 < D ≤ 0.1	Extreme disorder	VI	0.5 < D ≤ 0.6	Barely coordination
II	0.1 < D ≤ 0.2	Severe disorder	VII	0.6 < D ≤ 0.7	Primary coordination
III	0.2 < D ≤ 0.3	Moderate disorder	VIII	0.7 < D ≤ 0.8	Intermediate coordination
IV	0.3 < D ≤ 0.4	Mild disorder	IX	0.8 < D ≤ 0.9	Good coordination
V	0.4 < D ≤ 0.5	Near disorder	X	0.9 < D ≤ 1	Excellent coordination

#### Kernel density estimation

The Kernel density estimation is utilized for analyzing spatial imbalances and predicting trends in sample locations. This method effectively portrays the dynamic progression and trends in the CCD between LUEE and UER by allowing for the direct observation of smooth, continuous density curves. Owing to its robustness, it is extensively applied across various fields of research^62,63^.

12 $$f(x) = \frac{1}{Nh}\sum\limits_{i = 1}^{n} {K\left( {\frac{{X_{i} - x}}{h}} \right)}$$

where *N* signifies the aggregate count of sample observations, *X*_*i*_ denotes each observation that is independent and uniformly distributed; *x* corresponds to the average value; and *h* is indicative of the bandwidth, which is crucial in determining the smoothness of the Kernel density curve.

In alignment with existing literature, this study employs the Gaussian kernel function to investigate the evolving patterns of the CCD between LUEE and UER. The formula representing the Kernel function employed in this study is outlined below:

13 $$k(x) = \frac{1}{{\sqrt {2\pi } }}\exp \left( { - \frac{{x^{2} }}{2}} \right)$$

#### Convergence model

##### *σ*-convergence

Originally, *σ*-convergence describes the progressive reduction of regional per capita income discrepancies over time. This study applys the coefficient of variation as a tool to illustrate that the disparities in the CCD between LUEE and UER among 56 cities in the YRB, in relation to their deviation from the average, tend to diminish throughout the sample study period. The formula is outlined below:

14 $$\sigma_{t} = \frac{{\sqrt {\frac{1}{n}\sum\limits_{i = 1}^{n} {(F_{i,t} - \overline{F})^{2} } } }}{{\overline{F}_{t} }}$$

where *σ*_*t*_ represents the *σ-*convergence coefficient at time period *t*; *n* denotes the number of cities; *F*_*i,t*_ is the CCD value of city *i* at time *t*; and $\overline{F}_{t}$ is the average CCD value in time *t*.

##### *β*-convergence

*Β*-convergence implies that an economy’s improvement rate is inversely related to its initial level, indicating a catch-up trend among lagging economies towards more developed ones, ultimately leading to convergence. In this context, *β*-convergence suggests that regions with a lower CCD between LUEE and UER experience a higher growth rate compared to those with a higher CCD.

Furthermore, *β*-convergence is characterized by two primary forms: the absolute and the conditional. Absolute *β*-convergence occurs when regions converge without the need to control for additional influencing factors, whereas conditional *β*-convergence indicates convergence among regions after accounting for these factors. The specific model settings are outlined below:

15 $$\ln \left( {\frac{{F_{i,t + 1} }}{{F_{i,t} }}} \right) = \alpha + \beta \ln F_{i,t} + \eta_{t} + \lambda_{i} + \varepsilon_{it}$$

16 $$\ln \left( {\frac{{F_{i,t + 1} }}{{F_{i,t} }}} \right) = \alpha + \beta \ln F_{i,t} +\upgamma \sum\limits_{j = 1}^{n} {\ln Control_{it,j} } + \eta_{t} + \lambda_{i} + \varepsilon_{it}$$

Equations (15) and (16) respectively represent the absolute *β*-convergence and conditional *β*-convergence models. The term $\ln \left( {\frac{{F_{i,t + 1} }}{{F_{i,t} }}} \right)$ signifies the rate of increase in the CCD between LUEE and UER, calculated using its natural logarithm. *α* indicates a constant term, while the parameter *β*, acting as a convergence coefficient, suggests that if *β* < 0, the CCD between LUEE and UER exhibits convergence with statistical significance. *γ* denotes the coefficient of the control variables to be estimated. *η*_*t*_ denotes the time-related fixed effects, whereas *λ*_*i*_ reflects the fixed effects specific to various cities. *ε*_*it*_ corresponds to the equation’s random error term. The primary distinction between Eq. (15) and Eq. (16) is attributed to the incorporation of control variables, particularly focusing on the level of economic development (*Rpcgdp*), measured by real per capita GDP (based on 2003 constant prices); urbanization rate (*Urban*), gauged by the percentage of urban residents relative to the overall population; Industrial structure upgrading (*Upgrad*), indicated by the contribution of the tertiary sector to GDP, represented as a ratio; governmental behavior (*Govexp*), assessed by local government fiscal expenditure’s proportion to GDP; the level of technological innovation (*Innov*), represented by the number of patents granted in the city; and the degree of opening up (*Open*), demonstrated through the proportion of total imports and exports in relation to GDP.

##### Geodetector

Geodetector is a methodological tool designed to detect spatial stratification and heterogeneity in geographic phenomena, as well as to identify underlying driving factors^64^. Distinct from traditional models, the Geodetector is robust against multicollinearity, allowing for more effective identification of causal relationships between dependent and independent variables, thereby enhancing prediction accuracy. In this study, we utilize Geodetector’s dual approaches of the factor detection and interaction detection to examine the influence exerted by individual external factors on the CCD and the the effects of interactions among various influencing factors on the CCD. The explanatory power of an independent variable *X* is determined by its *q*-value, calculated using the following formula:

17 $$q = 1 - \frac{{\sum\limits_{h = 1}^{L} {n_{h} } \cdot \sigma_{h}^{2} }}{{n\sigma^{2} }}$$

where *L* denotes the layering within the independent variable *X*, *n* signifies the aggregate count of samples within the YRB, *σ*^2^ is the variance of the CCD (*Y*) between LUEE and UER, *n*_*h*_ and $\sigma_{h}^{2}$ are the number of units in stratum *h* and the variance of the dependent variable *Y*. The *q*-value fluctuates between [0,1], where elevated *q*-values suggest a more pronounced impact of the independent variable *X* on the variance observed in the dependent variable *Y*.

### Construction of the *indicator* system

#### Land use economic efficiency

In light of the critical importance of LUEE in improving the quality of economic growth^36^, this study employs the input–output ratios for assessing the economic capacity of urban land. The selected indicators are derived from four key input elements: land, capital, labor, and energy^65,66^. The output dimension primarily focuses on economic benefits, represented here by the value-added in secondary and tertiary industries (Table 2). Given the research’s focus on 56 cities in the YRB, the study incorporates the urban built-up area as the measure for land input. Investment in urban fixed assets is considered as the capital input, while employment in the secondary and tertiary sectors is used to quantify the labor input. The land area of the built-up zone is key in distributing and structurally modifying different types of urban functional areas, offering fundamental support for urban infrastructure and socio-economic activities. Moreover, energy, as a vital resource efficiency factor, in conjunction with labor and capital, significantly impacts a nation’s economic efficiency and competitiveness^54^. Therefore, this paper opts for these input–output indicators as an aggregate measure to assess LUEE.

**Table 2:** The indicator system for LUEE.

Indicator type	Description	Indicator layer	Unit
Input	Land	Urban built-up area	km^2^
	Labor	Employment in the secondary industry	person
		Employment in the tertiary industry	person
	Capital	Total investment in urban fixed assets	billions
	Energy	Total energy consumption	tons of standard coal
Output	Economic benefits	Value added of the secondary industry	billions
		Value added of the tertiary industry	billions

#### Urban ecological resilience

UER is fundamentally concerned with a system’s capacity to sustain functional stability under external disruptions and its ability to effectively reorganize, focusing on aspects like system resistance, adaptability, and recovery duration^67^. Accordingly, this study has curated 18 core indicators across the three dimensions of resistance, adaptability, and resilience, developing an all-encompassing assessment framework of indicators for UER (Table 3). In this context, resistance is defined as the city’s vulnerability and sensitivity to external shocks. To assess this, the study employs indicators such as industrial sulfur dioxide, industrial wastewater, and industrial fume (dust) emissions to depict the scale of industrial pollution; meanwhile, population density, annual average PM2.5, and energy carbon emissions serve as measures for the ecological environment’s carrying capacity. Adaptability is depicted by the capacity of cities to sustain ecosystem stability amidst disturbances. For this purpose, the study selects indicators such as the road sweeping and cleaning area, the general industrial solid waste comprehensive utilization rate, the centralized treatment rate of sewage treatment plants, and the harmless disposal rate of domestic waste. These metrics effectively demonstrate the urban ecosystem’s response efficacy. Additionally, the natural gas supply per capita and the drainage pipeline density in built-up areas serve to illustrate the ecosystem’s absorption capabilities. Resilience is conceptualized as the ecosystem’s potential to withstand disaster impacts, mitigate potential losses, and revert to its pre-disturbance state post-hazard exposure. This aspect is measured by indicators such as daily domestic water consumption per capita, the volume of domestic waste removed, and the land area per capita. These indicators collectively gauge the influence exerted by human activities on the ecological setting. The greening of urban areas is quantified through metrics like the green coverage rate, the rate of green land in built-up area, and the amount of green park area per capita.

**Table 3:** The indicator system for UER.

Components	Indicators	Properties	References
Resistance	Population density (people/km^2^)	−	^1,23,74^
	Industrial sulfur dioxide emissions (million tons/billion yuan)	−	^21,42,75^
	Industrial wastewater emissions (tons/billion yuan)	−	^21,42,75^
	Industrial fume (dust) emissions (tons/billion yuan)	−	^21,42^
	Annual average PM2.5 (mg/m^3^)	−	^42,52^
	Energy carbon emissions (million tons)	−	^75^
Adaptability	Road sweeping and cleaning area (10,000 m^2^)	+	^75^
	Natural gas supply per capita (m^3^/person)	–	^75^
	Harmless disposal rate of domestic waste (%)	+	^15,21^
	General industrial solid waste comprehensive utilization rate (%)	+	^1,15,21,42^
	Centralized treatment rate of sewage treatment plants (%)	+	^1,15,21,42^
	Drainage pipeline density in built-up areas (km/km^2^)	+	^75^
Resilience	Volume of domestic waste removed (million tons)	−	^59,75^
	Greening coverage rate in built-up areas (%)	+	^15,21,42^
	Rate of green land in built-up area (%)	+	^1,21^
	Per capita green park area (m^2^/person)	+	^1,15,59^
	Land area per capita (km^2^/million persons)	+	^21,59^
	Daily domestic water consumption per capita (liters)	+	^1,75^

When constructing comprehensive indicator systems for LUEE and UER, it is paramount to grasp the inherent correlations and disparities among certain indicators between these two systems, as this is crucial for uncovering their interactions. Taking labor input and population density as examples, there exists a degree of potential correlation between these two indicators. High population density areas typically imply a more abundant labor resource. Within the LUEE system, an ample labor force directly fosters various economic activities, thus serving as a positive input indicator for enhancing the LUEE. Conversely, within the UER system, high population density may indicate heightened pressure on ecosystems, thus population density is considered a key negative indicator of UER. Likewise, there exists some potential correlation between energy consumption and emissions of three wastes. In the LUEE system, energy serves as a pivotal driver for land development and utilization, constituting an important input indicator. However, within the UER system, emissions of three wastes signify the extent of industrial pollution, potentially leading to adverse environmental effects. Hence, when these indicators are comprehensively considered and analyzed, they can provide significant references for the sustainable development of cities, facilitating the promotion of a balance between economic development and ecological protection.

### Data sources

Considering data availability, this study selects 56 cities in the YRB, covering the period from 2003 to 2020, as research samples. Any missing data during this period are interpolated for completeness. The primary data sources include *the China City Statistical Yearbooks*, the EPS database, and the statistics yearbooks of each city. We employ MaxDEA software in conjunction with the super-efficiency SBM model to assess the input–output data of LUEE. Significantly, the total socio-economic energy consumption data are derived from an energy balance sheet, adjusted for standardization. All economic data are corrected for inflation to reflect actual values. Additionally, for the elevation mapping of the YRB, vector data are sourced from the Geospatial Data Cloud (https://www.gscloud.cn/). These data were processed using ArcGIS 10.8, involving meticulous delineation of the basin’s boundaries and subsequent cropping. The spatial resolution of the Digital Elevation Model (DEM) is 30 M, with data on primary rivers, major railroads, and urban locations obtained from the OpenStreetMap (OSM) open-source map website (https://www.openstreetmap.org/).

## Results

### Land use economic efficiency in Yellow River Basin

#### Temporal evolution of the LUEE

**Figure 4:**
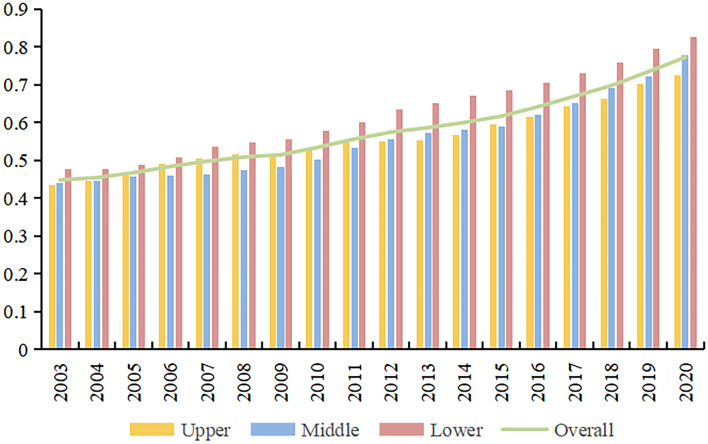
The average score of LUEE in the YRB.

Utilizing the super-efficiency SBM model, this study analyzes the LUEE across the overall YRB, including its sub-regions, from 2003 to 2020. The analysis reveals an ascending trend in the mean LUEE scores, as illustrated in Fig. 4. Notably, the descending order of LUEE scores is as follows: lower reaches, overall region, upper reaches, and middle reaches. The mean value of LUEE in the YRB as a whole rises from 0.4485 in 2003 to 0.7729 in 2020, marking a 72.31% increase. The lower reaches consistently demonstrated the highest efficiency, experiencing a steady rise except for a minor decline in 2004, culminating in a 73.31% growth by the end of the period. The mean value of LUEE in the upper and middle reaches displayed a staggered distribution pattern, oscillating around a 0.55 benchmark. Both regions exhibited a fluctuating but overall upward trend over time. Remarkably, the middle reaches surpassed the upper reaches in 2016, reaching third place with a 76.92% growth rate. This surge is likely attributed to the region’s recent focus on rational land resource utilization, significantly enhancing the LUEE.

#### Spatial evolution of the LUEE

**Figure 5:**
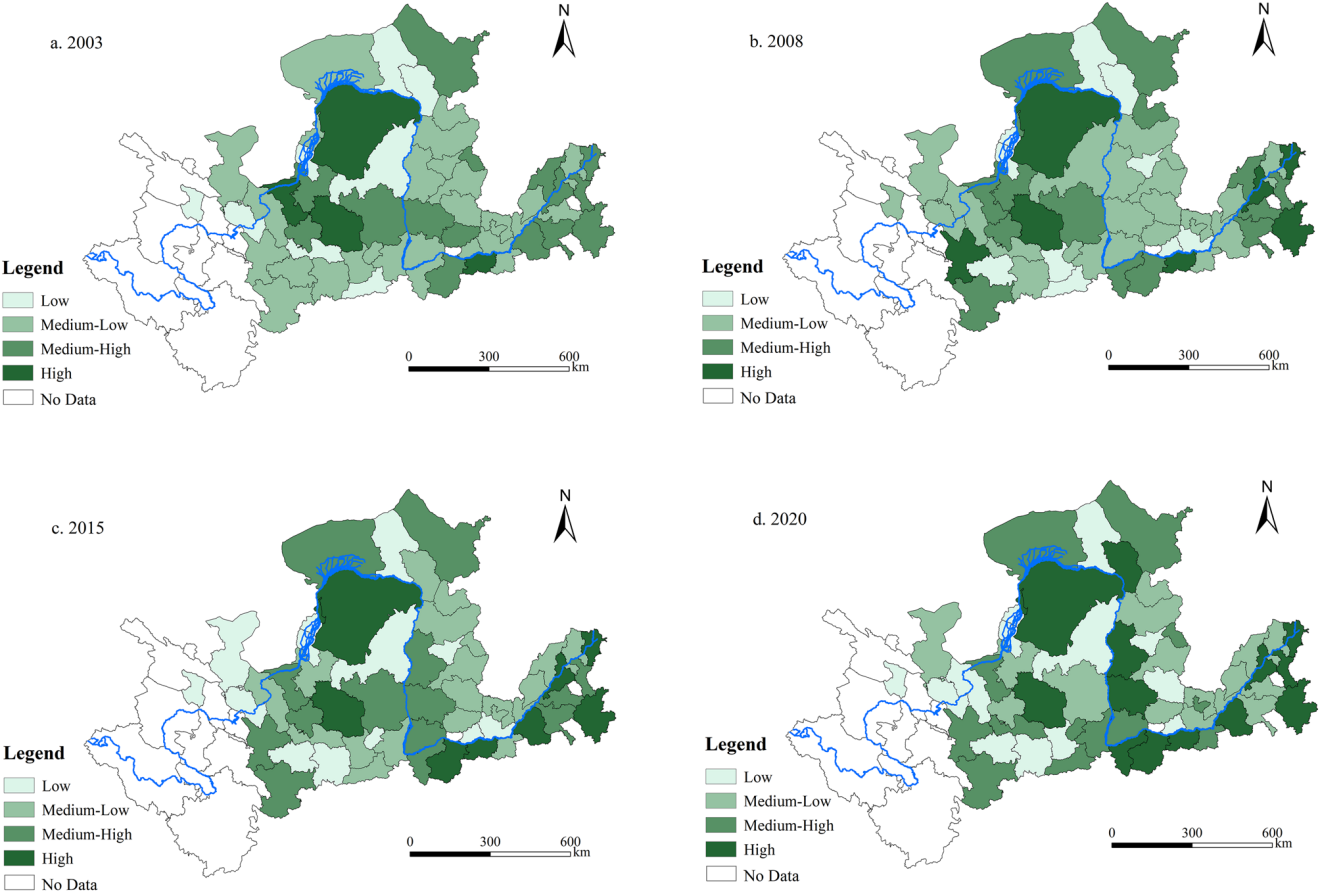
Spatial distribution of LUEE in the YRB.

This study employs ArcGIS 10.8 to create a spatial visualization vector map of LUEE in the YRB, aiming to delve into the spatial distribution traits and the progression of spatial configurations in different urban areas within the basin. Utilizing the “Jenks natural breakpoint”^68,69^, the study classifies the 56 cities in the YRB into four distinct LUEE categories: low, medium–low, medium–high, and high, as depicted in Fig. 5.

Figure 5 illustrates the progression of LUEE in the YRB for the years 2003, 2008, 2015, and 2020. It reveals a spatial imbalance in LUEE across the YRB. Cities with low and medium–low levels of LUEE predominantly cluster in the upper and middle reaches. In contrast, key cities like Zhengzhou, Ji’nan, Jining, Ordos, and Zhongwei in the lower reaches rank in the medium–high and high level categories. Notably, these cities in the lower reaches have shown a progressive increase in their LUEE levels over time. This trend likely stems from the lower reaches’ enhanced development resilience and potential relative to the upper and middle reaches. Additionally, a higher density of key cities and industrial parks in these regions contributes to the diversification and economic benefits in land use.

### Urban ecological resilience in Yellow River Basin

#### Temporal evolution of the UER

**Figure 6:**
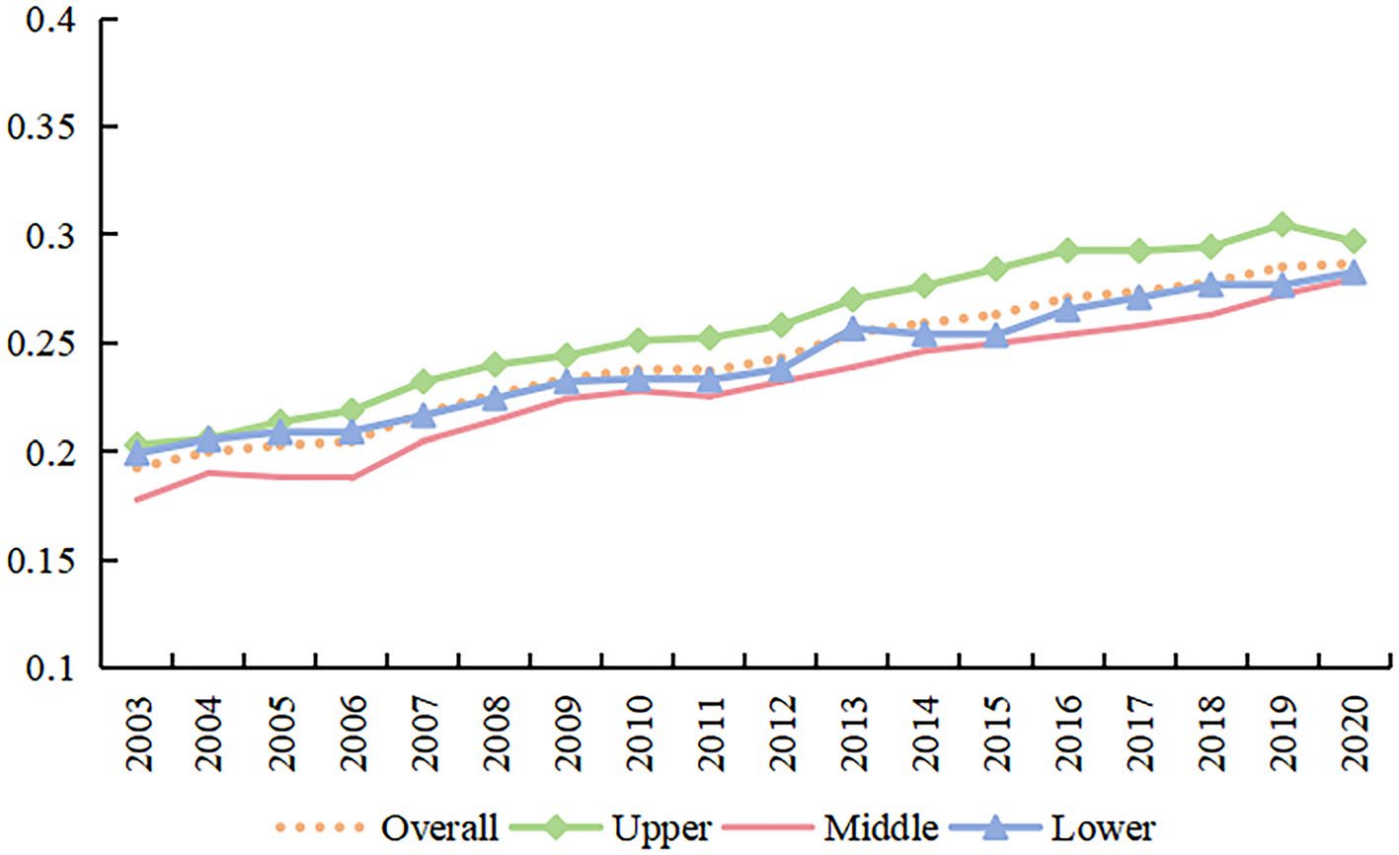
The average score of UER in the YRB.

This study utilized the entropy method to evaluate the UER. Figure 6 illustrates the trend of average UER scores across the YRB and its sub-regions from 2003 to 2020.

Specifically, the YRB’s overall UER score rose from 0.1926 in 2003 to 0.2867 in 2020, exhibiting a growth rate of 48.85%. In comparing the three regions, it was observed that the highest UER scores were in the upper reaches, with the lower and middle reaches trailing behind. All regions displayed a trend of gradual increase, albeit with some fluctuations. The upper reaches of the YRB, in particular, showed a relatively higher level of ecological resilience, primarily due to their relatively pristine and natural ecological environment. This environment contributes to ecological stability, fosters biodiversity, and enables the self-regulation of ecosystems, thereby enhancing their overall resilience.

#### Spatial evolution of the UER

**Figure 7:**
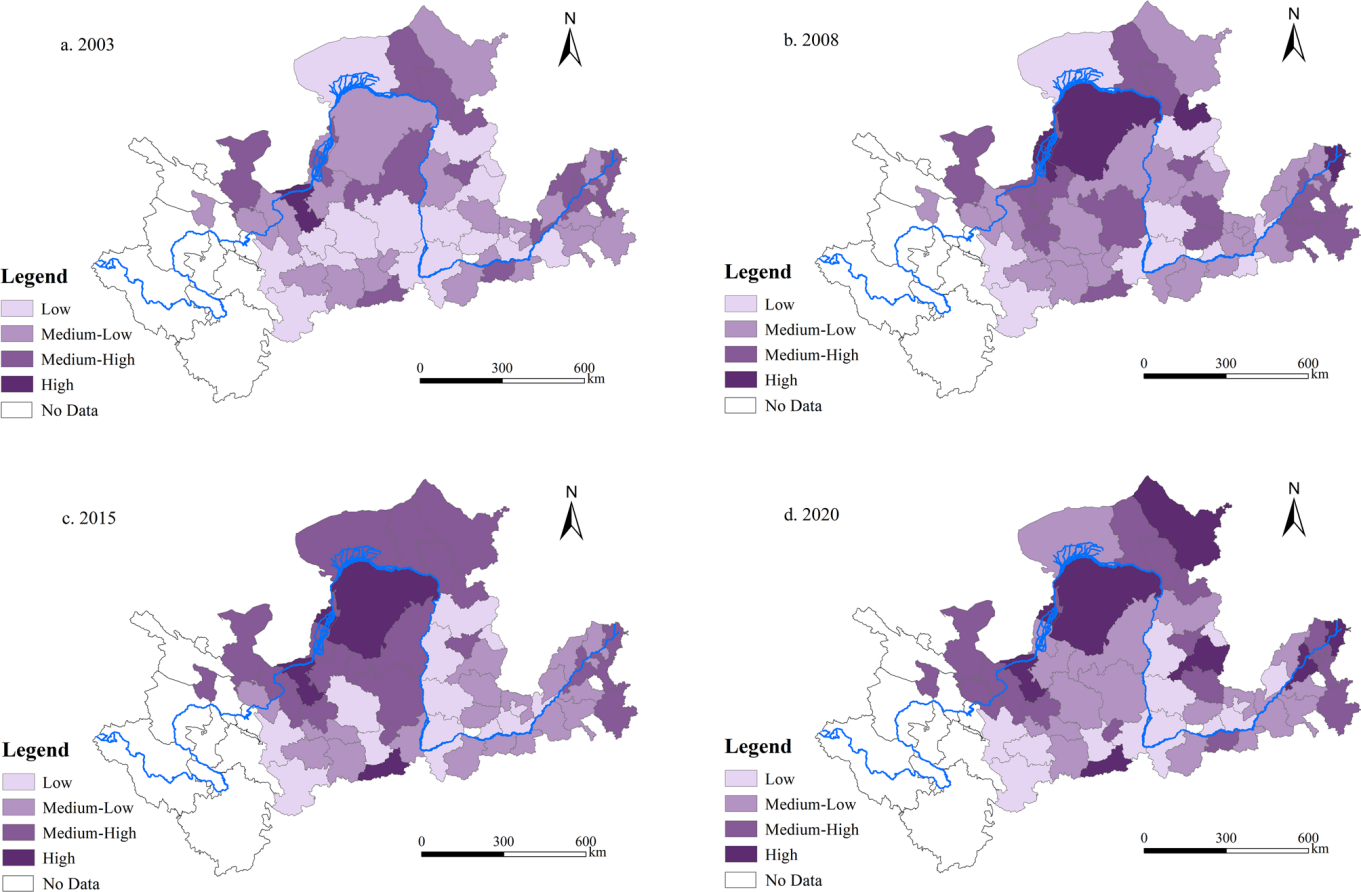
Spatial distribution of UER in the YRB.

To illustrate the spatial evolution of UER in the YRB, this study categorizes UER into four distinct categories: low, medium–low, medium–high, and high. This classification is achieved through the “Jenks natural breakpoint” technique implemented in ArcGIS 10.8, as depicted in Fig. 7.

According to Fig. 7, the UER scores display a spatial pattern characterized by “high in the upper reaches, low in the middle reaches, and high in the lower reaches”. Cities with higher levels of UER, such as Ji’nan, Jining, Zhengzhou, Hohhot, Zhongwei, and Ordos, are predominantly located in the upper and lower reaches. Over time, cities that maintain lower UER levels are primarily found in the middle reaches, exhibiting minimal changes in their numbers. Overall, the UER of the YRB has improved during the sample study period, showing a “high-low–high” pattern from the upper to the lower reaches. To enhance the overall UER of the YRB, focused efforts on pollution control and ecological restoration are crucial, particularly in the middle reaches.

### Coupling coordination effects in Yellow River Basin

#### Temporal evolution of the CCD

**Figure 8:**
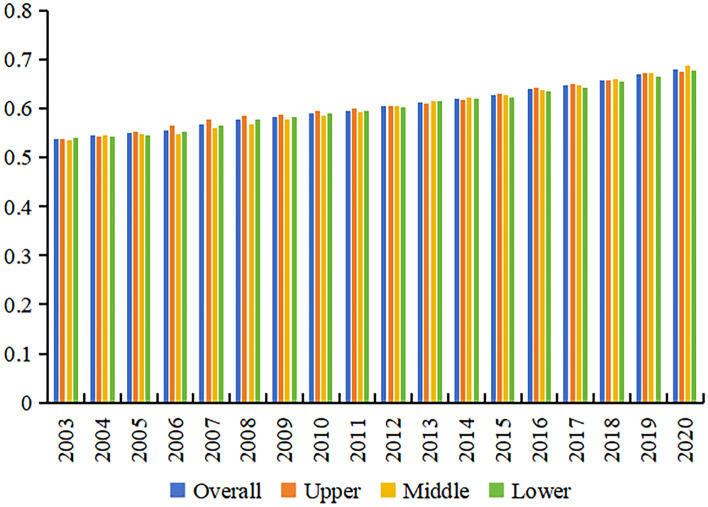
The average score of the CCD between LUEE and UER in the YRB.

This study employs the CCD model based on Eqs. (9)-(11) and a comprehensive evaluation system for LUEE and UER to assess their CCD in the YRB from 2003 to 2020. Figure 8 illustrates the average CCD between LUEE and UER in the YRB and its sub-regions.

Figure 8 reveals that from 2003 to 2020, the CCD between LUEE and UER in the YRB and its sub-regions consistently exhibited a rising trajectory. On a holistic level, the YRB’s average CCD between LUEE and UER displayed a slight but steady increase, ranging from 0.5371 to 0.6808. This range indicates a progression from just barely coordination to primarily coordination, highlighting potential areas for future improvement in both LUEE and UER within the YRB. To further enhance the overall CCD level of LUEE and UER in the YRB, efforts should be made to strengthen policy formulation and implementation, improve technological levels, increase public participation, and enhance cross-regional cooperation and coordination. Regionally, the lower reaches exhibit the highest CCD level across all sub-regions, with a growth rate of 25.12%, indicating a gradual enhancement. The upper reaches show a slow upward trend in the CCD between LUEE and UER, exhibiting an average annual increase of merely 1.37%, and the evolution of the coupling coordination type remains relatively stable. Despite having the lowest CCD level in the YRB, the middle reaches are at the forefront in growth rate, registering a significant increase of 29.15%. This indicates a progressively strengthening coordination between LUEE and UER in the region.

**Figure 9:**
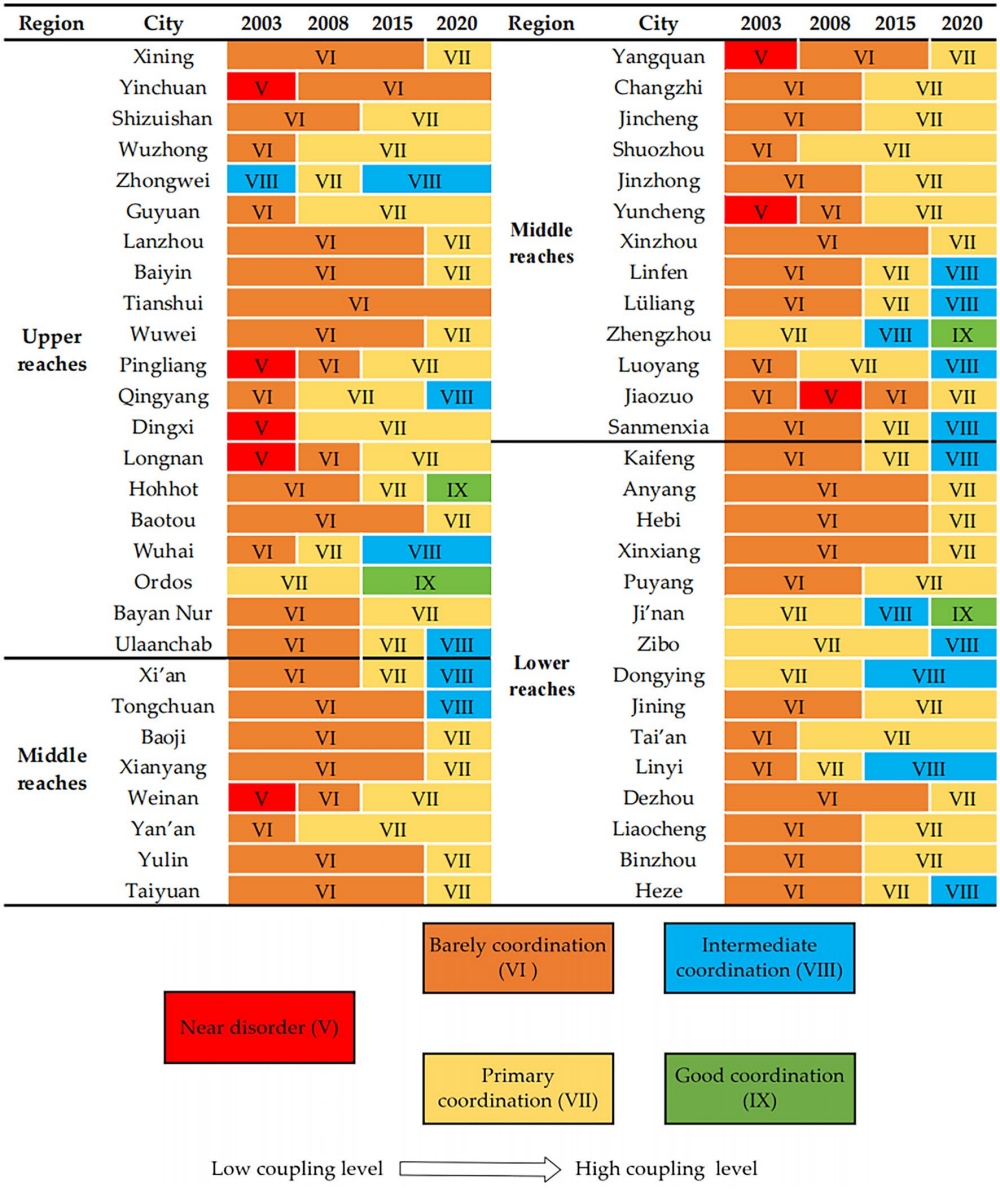
Classification of the CCD in YRB in 2003, 2008, 2015 and 2020.

To delve into the detailed evolutionary trend of CCD, this study systematically analyzed the CCD of 56 cities for the years 2003, 2008, 2015, and 2020. This analysis is visually represented through varying colors for different types of coupling coordination, as shown in Fig. 9.

Figure 9 reveals that the CCD between LUEE and UER for these cities generally progressed through stages from “near disorder” to “barely coordination”, “primary coordination”, “intermediate coordination”, and finally “good coordination”. Over time, both systems have improved to varying degrees. Generally, the initial CCD levels of cities in the lower reaches were at least “barely coordination”, higher than those in other reaches. Notably, Ji’nan City advanced from “primary coordination” to “good coordination”, marking a significant deepening in coupling association. In the upper reaches, the span of coupling coordination types was more extensive. Cities like Hohhot, Qingyang, Pingliang, Dingxi, Longnan, and Wuhai achieved a three-level jump by the end of the period. Hohhot and Erdos were particularly prominent, eventually reaching the “good coordination” level. Some cities in the middle reaches also experienced an increase in the span of coordination grades, with an overall upward trend. Cities like Zhengzhou, Weinan, Yangquan, Yuncheng, Linyi, and Lüliang evolved three coordination levels, from “near disorder” and “barely coordination” to “primary coordination” and “intermediate coordination”. Except for Jiaozuo City, which saw a slight decrease in CCD in 2008, all other cities showed a clear increasing trend in the CCD between LUEE and UER during the sample study period. Overall, the CCD level of cities in the YRB generally shows an upward trend, and the coupling type shifts to better over time^52^. This progress is attributed to cities’ rigorous control over the increase in construction land, improvements in LUE, intensified pollution control efforts, and refined environmental policy frameworks^70^. These initiatives not only promote economic efficiency and sustainability in urban land use but also strengthen UER, thus advancing the twin goals of economic benefit and environmental protection.

#### Spatial evolution of the CCD

**Figure 10:**
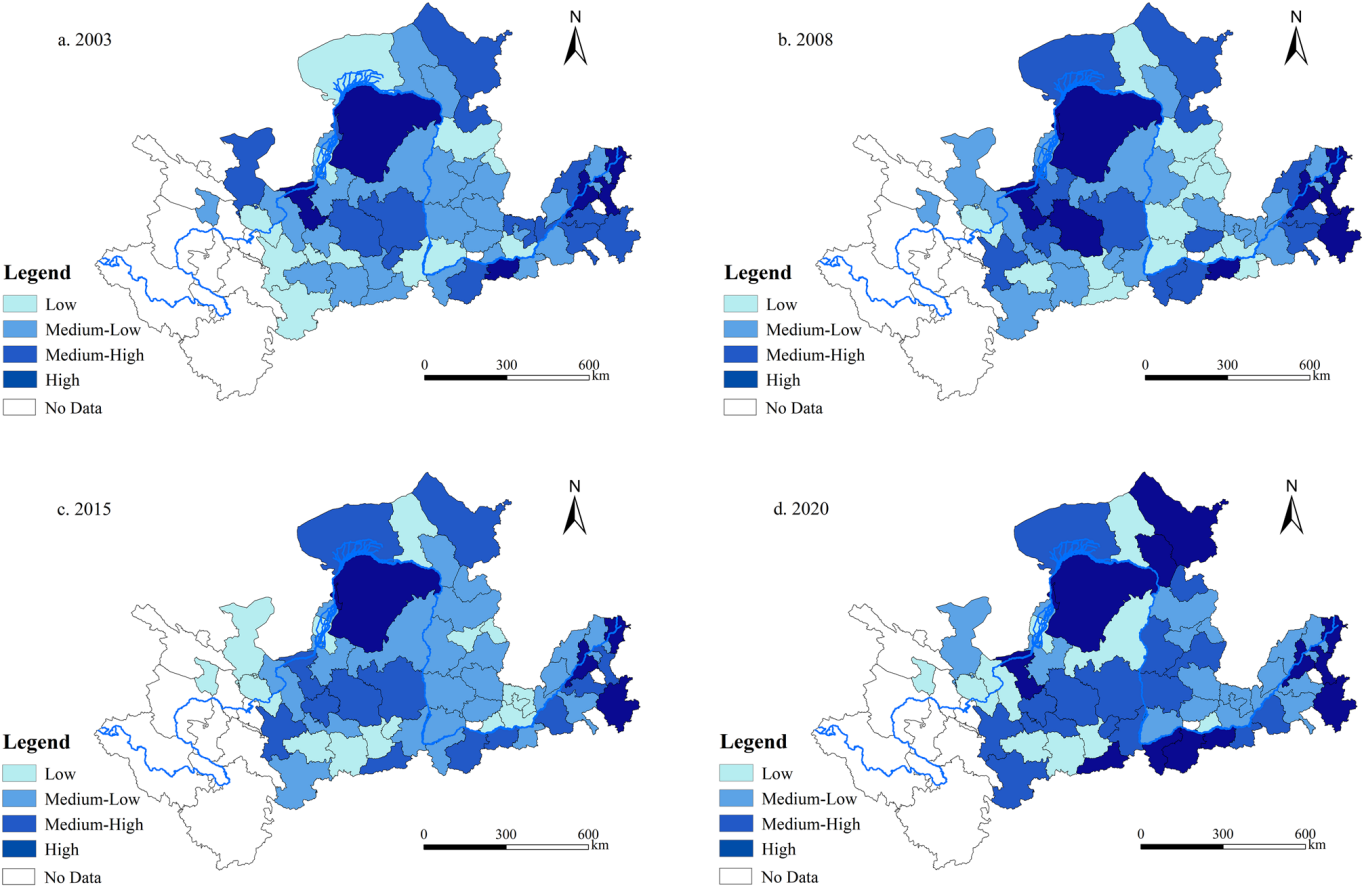
Spatial distribution of the CCD between LUEE and UER in the YRB.

This paper aims to study the spatial trends in the CCD between LUEE and UER more intuitively and accurately. To this end, it utilizes the “Jenks natural breakpoint” technique implemented in ArcGIS 10.8 to geospatially visualize the CCD of these factors in 2003, 2008, 2015, and 2020. Figure 10 depicts the CCD of 56 cities in the YRB at various time points, highlighting their differentiated development characteristics.

As evidenced by Fig. 10, there is a noticeable geographical imbalance in the CCD between LUEE and UER in the YRB. During 2003, 2008, 2015, and 2020, areas with high levels of CCD predominantly include key cities such as Ji’nan and Hohhot, and cities like Ordos, Zhongwei, and Linyi in the upper and lower reaches, demonstrating a “regional aggregation effect”. This phenomenon may be attributed to these key cities acting as primary drivers in enhancing LUEE and UER in the YRB. Their success is likely due to abundant land resources, green technological innovations, well-developed infrastructure, and favorable national policies. Conversely, areas with medium–low and low levels of CCD, including cities like Xianyang, Xinzhou, Taiyuan, and Yangquan, predominantly located along the middle reaches, exhibited minimal typological changes during the sample study period. From these analyses, it is evident that the main spatial distribution characteristics of CCD between LUEE and UER in the YRB follow a distinct trend of being “high at both ends and low in the middle”.

#### Spatial autocorrelation analysis

To investigate the spatial correlation of the CCD, this study calculates the Global Moran’s I for CCD in 56 cities from 2003 to 2020. The calculations are based on a geographic neighborhood weight matrix, because the geographic neighborhood weight matrix can well reflect the adjacency relationship between spatial units, thereby helping us to understand more deeply the interactions between spatial units. Furthermore, this analysis utilizing Stata 17.0 software, and include significance testing. Table 4 provides a detailed presentation of the results.

**Table 4:** Global Moran’s I for the CCD.

Year	Global Moran’s I	*P*-value	Z-value
2003	0.315	0.000	3.899
2004	0.292	0.001	3.378
2005	0.282	0.001	3.388
2006	0.307	0.000	3.646
2007	0.325	0.000	3.821
2008	0.371	0.000	4.348
2009	0.349	0.000	4.166
2010	0.269	0.001	3.218
2011	0.384	0.000	4.552
2012	0.361	0.000	4.271
2013	0.391	0.000	4.617
2014	0.430	0.000	5.021
2015	0.415	0.000	4.903
2016	0.484	0.000	5.761
2017	0.550	0.000	6.487
2018	0.567	0.000	6.715
2019	0.506	0.000	5.975
2020	0.599	0.000	6.996

According to the data presented in Table 4, the Global Moran’s I value for the CCD is consistently above 0.28, with |Z|> 1.96, thereby surpassing the threshold of 1% significance. This suggests a positive spatial autocorrelation at the overall level within the YRB. Specifically, cities with high values in these metrics are typically surrounded by other high-value cities, while low-value cities are adjacent to similar low-value cities. Although there are individual yearly decreases, the absolute value of the Global Moran’s I generally shows a rising trend.

**Figure 11:**
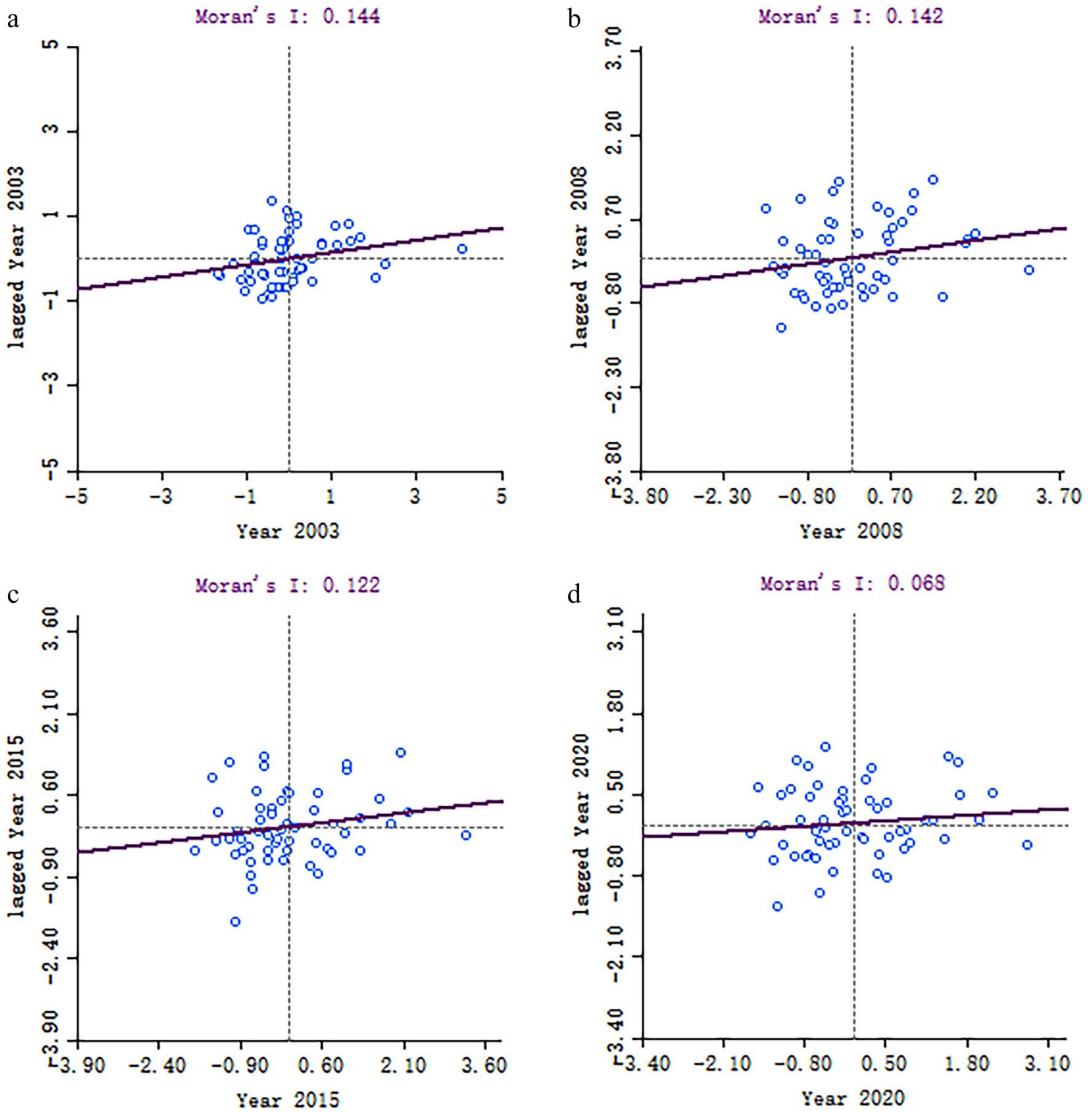
Moran’s I scatterplot for CCD in 2003, 2008, 2015 and 2020.

**Figure 12:**
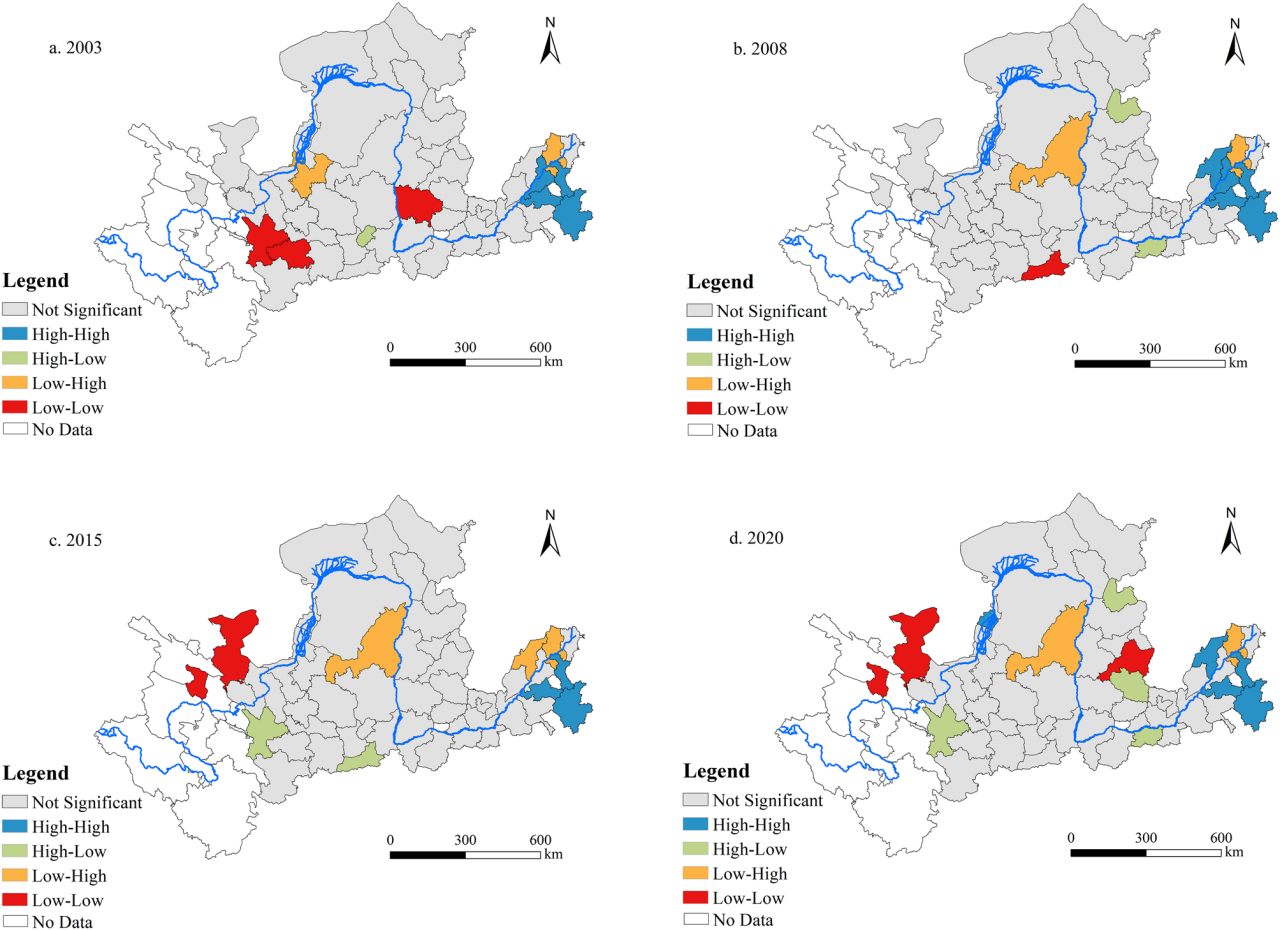
Local indicators of spatial association (LISA) aggregation map.

To provide a clearer understanding of the CCD’s local spatial correlation characteristics and analyze the significance of local spatial clustering and regional localization of cluster types, this study selects the years 2003, 2008, 2015, and 2020. Local Moran scatter plots and local spatial autocorrelation (LISA) cluster maps at a significance level of P < 0.05 are created using ArcGIS 10.8 and Geoda software, respectively. The results of these analyses are shown in Figs. 11 and 12.

The results depicted in Fig. 11 show that the absolute values of the Local Moran’s I for the years 2003, 2008, 2015, and 2020 are all greater than zero. This suggests that there was a consistent positive spatial correlation in the overall CCD during these years, with a noticeable degree of spatial clustering. However, regional spatial integration remains relatively weak, as the autocorrelation between each city and its neighboring cities within the region has gradually diminished over time. Of particular significance is the conclusion of the sample study period, during which the local spatial autocorrelation index of the CCD becomes distributed among all quadrants, indicative of the CCD’s most diminished spatial spillover effect. This trend may be attributed to the fact that the YRB extends across the eastern, central, and western parts of China. The significant differences in land development and utilization patterns, industrial structures and development strategies, resource endowments, and ecological environmental protection efforts across the basin have led to a low level of synergy between the LUEE and the UER of the cities, thus hindering the formation of an effective cohesion.

Figure 12 illustrates the LISA clustering distribution maps for 56 cities in the YRB across the years 2003, 2008, 2015, and 2020. These maps exhibit four distinct clustering patterns: “high-high”, “high-low”, “low–high”, and “low-low”. The “high-high” (“low-low”) clustering pattern signifies that a city with a high (low) value for the study variable is encircled by other cities also exhibiting high (low) values, reflecting a positive spatial correlation with its neighboring areas. On the contrary, the “high-low” (“low–high”) clustering pattern suggests that a city with a high (low) value for the study variable is surrounded by areas with low (high) values, demonstrating a negative spatial correlation with its surrounding areas.

An analysis of the selected years reveals that “high-high” clustering cities are predominantly found in the lower reaches. This may be attributed to the lower reaches leveraging the diffusion effects of green technological innovations and the spatial spillover effects from the flow of production factors, which collectively foster the interconnected growth of neighboring cities and contribute to forming regional growth poles for coordinated development. Cities including Dingxi, Zhengzhou, Xi’an, and Shuozhou exhibited “high-low” clustering characteristics during the sample study period. This pattern suggests that these cities maintain high levels of CCD between LUEE and UER, while their adjacent cities exhibit lower levels of CCD. This phenomenon may be due to the insufficient spatial spillover effects of these cities, resulting in their urban advantages failing to effectively drive the synergistic development of neighboring cities. Specific economic status and policy favoritism have contributed to the increase in the CCD between LUEE and UER in these cities, while neighboring cities have not been able to reach the same level due to relatively insufficient resources and policy support. Additionally, the economic activities and policies of high-level cities may have negative external effects on neighboring cities, such as resource crowding and pollution spreading, further widening the gap in coupling levels. Conversely, cities like Yulin, Wuzhong, and Binzhou fall into the “low–high” clustering category, displaying a relatively dispersed spatial distribution pattern. The “low-low” clustering cities are predominantly situated in the upper and middle reaches, characterized by a more dispersed spatial distribution. Overall, to achieve the ambitious goal of transitioning the YRB towards an advanced stage of excellent coordination type, it is crucial to fully harness the technology diffusion and leading influence of the lower reaches, thereby enhancing the CCD level between the two systems.

### Dynamic evolution trend in Yellow River Basin

**Figure 13:**
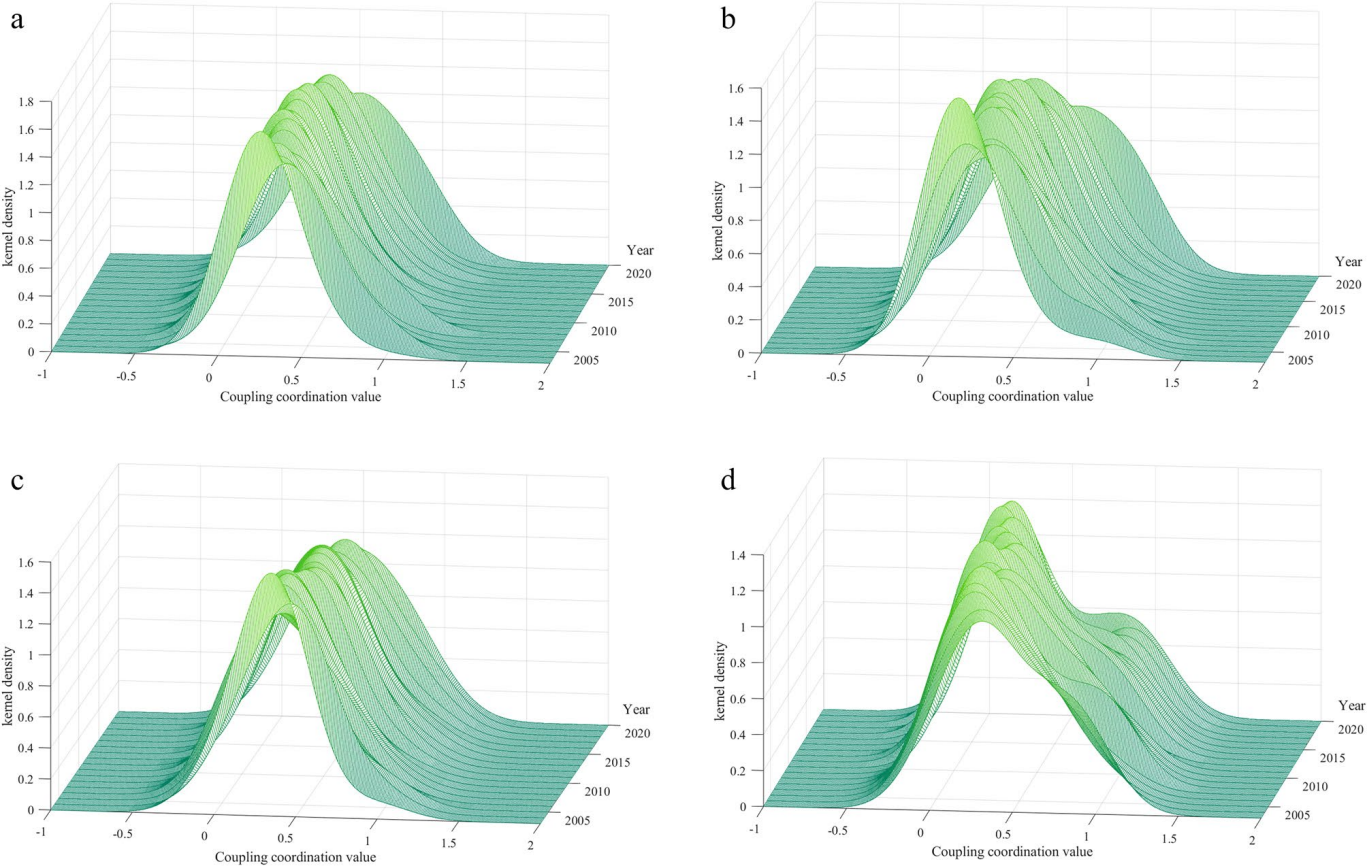
Dynamic evolution trend of CCD in the YRB.

For a thorough examination of the dynamic progression of the CCD between LUEE and UER, this paper employs Matlab 2022b software to create three-dimensional Kernel density estimation plots. These plots characterize the distribution features of the YRB and its sub-regions, focusing on key attributes like distribution location, pattern, and polarization trend. The specific Kernel density estimation plots are depicted in Fig. 13.

Overall, Fig. 13a presents a three-dimensional perspective of the Kernel density for the overall YRB. The main characteristics are as follows: First, the center point of the Kernel density function gradually shifts to the right during the sample study period, indicating an improvement in the CCD. Second, the apex of the Kernel density curve exhibits a trend of increasing initially and then decreasing, with the main peak’s breadth starting off narrow and later expanding. This suggests that there is no significant fluctuation in the CCD of cities within the YRB during the sample study period. Third, the number of wave peaks consistently remains at one from 2003 to 2020, indicating an absence of polarization in the CCD across the YRB. In recent years, the government has curtailed the over-exploitation of natural resources in the YRB through measures such as ecological compensation mechanisms and environmental protection policies to ensure the sustainability of economic activities. In overall land use planning, ecological factors are fully considered, which enhances the efficiency of land resource utilization while preserving the integrity of the ecosystem. The implementation of these policies has effectively mitigated potential conflicts between economic development and ecological protection, resulting in a relatively small gap in the CCD between LUEE and UER in the YRB.

Regionally, Fig. 13b, c, d detail the dynamic progression of the CCD between LUEE and UER in the three sub-regions of the YRB. From Fig. 13b, the Kernel density curve of the upper reaches generally trends rightward, indicating a continual improvement in the CCD. The main peak’s height undergoes an “N”-shaped fluctuation. Consistently, the number of wave peaks remains at one, signifying the absence of polarization. Figure 13c shows that there is a continual rightward shift in the Kernel density curve within the middle reaches, reflecting a gradual increase in the CCD over time. The elevation of the main peak remains relatively unchanged, indicating a stable level of the CCD. The middle reaches predominantly exhibit a single peak phenomenon, with no apparent polarization in the distribution of the CCD. In Fig. 13d, the lower reaches’ Kernel density curve exhibits pronounced “right-tail” characteristics, signifying a rising trend in the CCD. The main peak’s vertical elevation shows an upward trend, suggesting a centralized trend in the CCD between LUEE and UER. The Kernel density curve displays a double-peak phenomenon, with a stable height difference between the main and side peaks, indicating a distinct gradient effect in the CCD, this suggests a more pronounced bifurcation trend in the lower reaches. In general, only the cities in the lower reaches of the YRB have a bifurcated situation, and there is no obvious polarization phenomenon in the whole and the rest of the region. The reason for this may be due to the fact that some of the cities in the lower reaches have a head start in terms of geographic location and resource distribution, coupled with the difference in the sequence of capital investment and the regional orientation of the government’s supportive policies in the early stages, which has led to the existence of a more obvious difference between the cities in the lower reaches^71^.

### Convergence analysis in Yellow River Basin

#### σ-convergence

**Figure 14:**
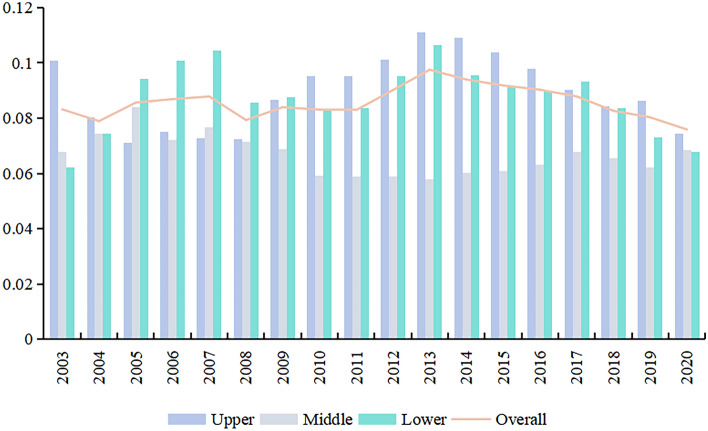
*σ*-convergence results.

Building on the analysis of the CCD’s dynamic evolutionary trend, this study employs a convergence model to calculate the *σ*-convergence index for the overall YRB as well as its sub-regions. This approach is intended to offer more profound understanding of the convergence traits of the CCD, with the specific *σ*-convergence results presented in Fig. 14.

As a whole, the *σ*-convergence coefficient exhibits a fluctuating downward trend from 2003 to 2020, decreasing from 0.0832 in 2003 to 0.0759 in 2020, a reduction of 8.73%. This suggests the presence of a *σ*-convergence trend in the CCD in the YRB. Regionally, the lower reaches’ coefficient of variation follows an “M”-shaped pattern of “rising-falling-rising-falling”, ending with a higher convergence coefficient than at the beginning, increasing by 9.05%. This suggests the absence of *σ*-convergence in the lower reaches, instead showing characteristics of dispersion, which aligns with the conclusions drawn from the Kernel density analysis earlier. The *σ*-convergence curve for the upper reaches displays a significant decreasing trend, with a decline of 26.06%, indicating the most pronounced convergence effect among the regions. The *σ*-convergence curve in the middle reaches exhibits a fluctuating, slightly increasing trend, with the *σ*-convergence value rising from 0.0677 in 2003 to 0.0683 in 2020, an increase of only 0.92%, signifying a less pronounced dispersion effect.

#### β-convergence

Building upon the previously mentioned absolute *β*-convergence and conditional* β*-convergence models, and addressing potential concerns of endogeneity, this study constructs a dynamic panel model and applies the System Generalized Method of Moments (Sys-GMM) estimation to test the *β*-convergence effect on the CCD between LUEE and UER in the YRB. The *β*-convergence estimation results for the CCD are illustrated in Table 5.

**Table 5:** *β*-convergence results for the CCD.

Variable	(1)	(2)	(3)	(4)	(5)	(6)	(7)	(8)
	Overall		Upper		Middle		Lower	
*β*	− 0.174***	− 0.446***	− 0.257***	− 0.267***	− 0.184***	− 0.418***	− 0.256**	− 0.475***
	(0.021)	(0.055)	(0.054)	(0.063)	(0.033)	(0.078)	(0.094)	(0.134)
*Rpcgdp*		0.027***		0.008		0.047***		0.047***
		(0.009)		(0.008)		(0.017)		(0.018)
*Urban*		− 0.005		− 0.028**		− 0.017		− 0.058**
		(0.014)		(0.013)		(0.017)		(0.028)
*Upgrad*		0.072***		0.057***		0.141***		0.135
		(0.020)		(0.014)		(0.043)		(0.089)
*Govexp*		0.059		0.030		− 0.001		0.0833
		(0.043)		(0.028)		(0.128)		(0.151)
*Open*		− 0.075*		− 0.006		− 0.032		− 0.166***
		(0.043)		(0.016)		(0.079)		(0.058)
*Innov*		0.003***		0.002		0.001		0.000
		(0.001)		(0.001)		(0.001)		(0.001)
Constant	− 0.072***	− 0.508***	− 0.148***	− 0.104	− 0.099***	− 0.640***	− 0.146**	− 0.369
	(0.010)	(0.147)	(0.034)	(0.072)	(0.021)	(0.212)	(0.053)	(0.339)
Time fixed effects	Yes	Yes	Yes	Yes	Yes	Yes	Yes	Yes
City fixed effects	Yes	Yes	Yes	Yes	Yes	Yes	Yes	Yes
N	1008	1008	360	360	378	336	270	270
R^2^	0.021	0.051	0.054	0.063	0.033	0.078	0.163	0.134
Cities	56	56	20	20	21	21	15	15

Table 5 displays the absolute *β*-convergence estimation results for the YRB as a whole and for its upper, middle, and lower reaches in columns (1), (3), (5), and (7). Meanwhile, the corresponding estimation results of the conditional *β*-convergence are depicted in columns (2), (4), (6), and (8). Analysis of the odd-numbered columns reveals that the *β*-values for the entire basin and its sub-regions are significantly negative. This suggests that there is a discernible trend of absolute* β*-convergence in the CCD, with a trend towards convergence at their respective steady-state levels. The even-numbered columns show that the *β*-values for these regions are less than zero and pass the 1% statistical significance test, providing evidence for the existence of a conditional *β*-convergence mechanism in these areas. Additionally, a comparison reveals that the conditional *β*-convergence coefficients, in terms of their absolute values, exceed those of the absolute *β*-convergence in the YRB and its sub-regions. This suggests that, when factors such as the level of economic development, urbanization rate, Industrial structure upgrading, government behavior, level of technological innovation, and the degree of opening up are taken into account, there is an accelerated rate of convergence in the CCD. The order of convergence speed, from fastest to slowest, is as follows: lower reaches > overall region > middle reaches > upper reaches. Overall, the addition of relevant control variables significantly accelerates the CCD’s convergence towards their respective steady-state levels^21,72^. The YRB and its sub-regions display significant variations in industrial structure upgrading, resource factor allocation, technological innovation, and government policy support^73^. These differences result in varying speeds of conditional *β*-convergence across regions. However, considering the overall convergence characteristics, these conditional factors collectively expedite the convergence of the CCD between LUEE and UER. This acceleration contributes to the balance and stability of urban systems, fostering the economic benefits associated with land utilization, and supporting the healthy development of ecosystems.

### Driving factors in Yellow River Basin

In order to explore the underlying causes behind the fluctuations in the CCD between LUEE and UER in the YRB, we designate the CCD as the dependent variable. Utilizing the “Jenks natural breakpoint” method for categorizing factor variables^63,64^, this paper, in line with existing research^65–68^ and considering the region’s actual developmental context, selects indicators such as topographic relief (*X*1), population density (*X*2), distance to the provincial capital city (*X*3), industrial structure upgrading (*X*4), government fiscal budget expenditure (*X*5), and urbanization level (*X*6) as key detect factors. This approach aims to reveal the drivers influencing the CCD in the YRB.

**Figure 15:**
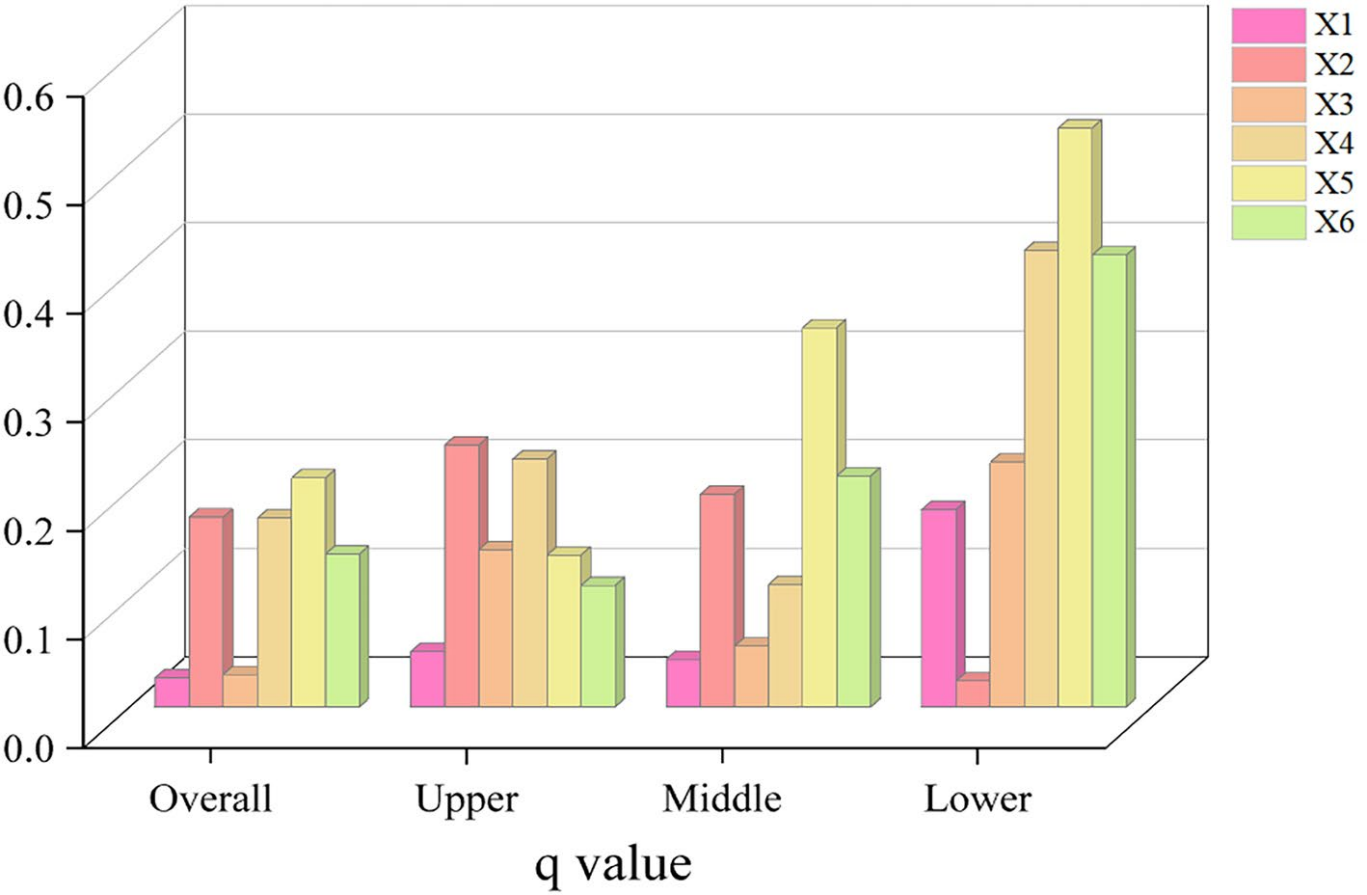
Geodetector factorization results.

Figure 15 presents the detection power (*q*-value) of each driving factor for the CCD between LUEE and UER in the YRB and its sub-regions. The impact of every distinct factor on the CCD becomes more pronounced as the *q*-value increases. Across the entire study area, government fiscal budget expenditure has the most significant independent driving effect on CCD (*q* = 0.211), while topographic relief has the smallest (*q* = 0.027). When breaking down the region into upper, middle, and lower reaches, the driving factors for CCD vary, with population density (*q* = 0.241) being the primary driver in the upper reaches and government fiscal budget expenditure (*q* = 0.349 and 0.533) in the middle and lower reaches, respectively. Overall, government fiscal budget expenditure (*X*5) emerges as the most influential factor for CCD across the regions studied, becoming the key driver of the CCD in the YRB. Government policy orientation is pivotal in securing urban economic development and ecological protection, maximizing economic benefits, and enhancing urban sustainability through improved land use planning, environmental protection, and ecological restoration, supported by financial investment.

**Figure 16:**
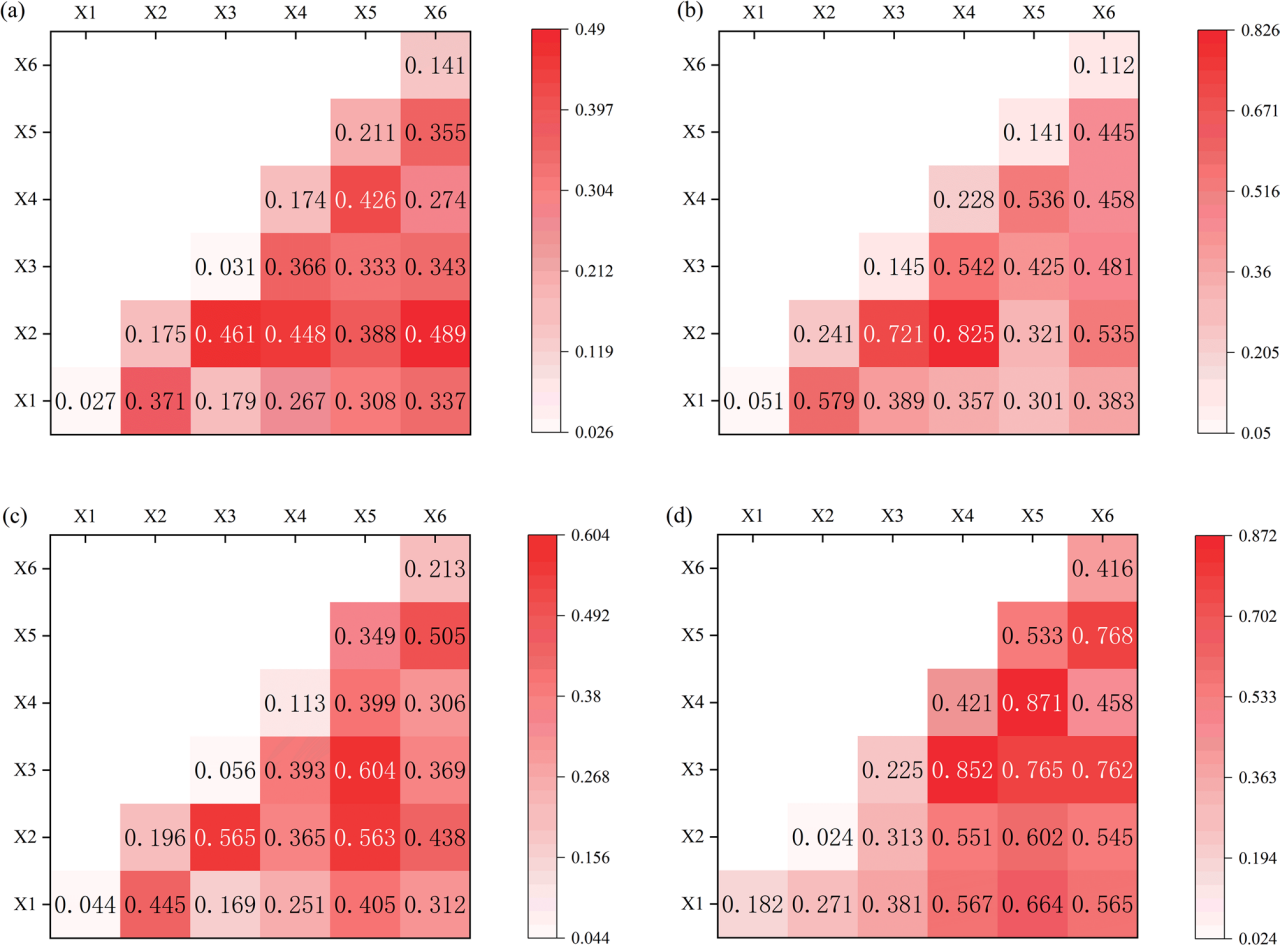
Driver interaction results.

This study analyzes the interaction effects among six driving factors impacting the CCD between LUEE and UER in the YRB, as demonstrated in Fig. 16. The findings suggest that the interactions between these factors are more significant than the influence of each factor independently. Specifically, across the YRB, population density and urbanization level emerge as the most influential on the CCD (*X*2 ∩ *X*6 = 0.489), while topographic relief and distance to the provincial capital city have the least effect (*X*1 ∩ *X*3 = 0.179). The interactions among the other factors average around a *q*-value of 0.3. Regionally, distinct dynamics are observed: in the upper reaches, the synergistic effect of population density and distance to the provincial capital city is the key driver for CCD (*X*2 ∩ *X*3 = 0.721), whereas the interaction between topographic relief and government fiscal budget expenditure has minimal influence (*X*1 ∩ *X*5 = 0.301). In the middle reaches, distance to the provincial capital city and government fiscal budget expenditure significantly impact CCD (*X*3 ∩ *X*5 = 0.604), with topographic relief and distance to the provincial capital city being least impactful (*X*1 ∩ *X*3 = 0.169). Conversely, in the lower reaches, the industrial structure upgrading and government fiscal budget expenditure predominantly shape CCD (*X*4 ∩ *X*5 = 0.871), while topographic relief and population density exert the least influence (*X*1 ∩ *X*2 = 0.271). This phenomenon can be attributed to the industrial structure in the lower reaches, which is still characterized by high energy consumption and intensive inputs, facing the dual challenges of resource depletion and ecological strain. To address these ecological challenges, the government has increased fiscal budget expenditure to adjust and optimize the industrial structure, introducing high-value-added and innovative technology industries. This not only facilitates an effective upgrade of the industrial structure but also stimulates a rise in economic activity. Consequently, the interplay of these two factors significantly impacts the CCD between LUEE and UER, thus facilitating harmonious and enduring advancement of both the regional economy and societal aspects.

## Discussion

In the face of global ecological challenges, including rampant urban sprawl, varied climate change impacts, excessive pollution emissions, and spatial structural imbalances, the pursuit of sustainable urbanization is critical. This necessitates a well-thought-out urban land layout to substantially reduce ecological harm and resource wastage. Enhancing urban ecosystems’ resilience to withstand internal and external disturbances is essential for sustainable urban development. In this context, LUEE and UER have been the focus of many studies in recent years. However, most previous studies have assessed LUEE or UER individually, ignoring the intrinsic interrelationships between them^21,34,67^. Given this situation, our study introduces the CCD model to systematically explore the coupling coordination relationship between LUEE and UER. Notably, Zhang et al.^36^ conducted a similar study, in which they found that the construction of green manufacturing systems lagged behind LUEE and the CCD between the two was characterized by phased development. In addition, Su et al.^77^ measured the coupling level of LUE and ecological environmental quality through a CCD model and found that the two systems exhibited interaction and significant spatial and temporal heterogeneity. Likewise, Jing et al.^48^ and Wu et al.^78^ reached similar conclusions, which further validated our findings. In our study, we found that the CCD between LUEE and UER was higher in the lower reaches of YRB than in other regions. To alleviate such regional differences, policy guidance should be strengthened, spatial layouts optimized, and regional cooperation mechanisms implemented to facilitate resource sharing, complementary advantages, and synergistic development among cities in the YRB, thereby enhancing the level of coupling coordination between LUEE and UER across the entire basin.

In exploring the driving factors of the CCD between LUEE and UER, government fiscal budget expenditure emerges as the most significant independent influencing factor. This differs from the findings of Jing et al.^48^, who identified population density as the most significant explanatory factor for the CCD between LUE and ecological environment quality. The disparity may stem from the highly uneven population distribution across different regions of the country, resulting in varied demands and pressures on land resources. In contrast, the YRB, a region of special geographic and ecological importance, sees its land use and ecological protection more significantly influenced by government policies and fiscal expenditures. Thus, government financial budget expenditure becomes the most key factor. Our study also reveals that the combined influence of both factors is greater than the influence of each factor individually. This finding aligns with the conclusions of Zhang et al.^79^ and Shen et al.^80^. Our results indicate that the interaction between industrial structure upgrading (*X4*) and government fiscal budget expenditure (*X5*) on CCD is most pronounced in the lower reaches of the YRB, providing a new perspective and basis for policy formulation in the region.

This study employed the CCD model to investigate the interaction between LUEE and UER in the YRB. It advances previous research by providing a more comprehensive dissection of the spatial and temporal distribution features and the inherent mechanisms of CCD between LUEE and UER. Nonetheless, our research has its limitations. Firstly, in developing the indicator system, we aimed to balance comprehensiveness, systematicity, and operability in selecting indicators. However, due to data availability constraints, there were inevitable shortcomings in covering essential indicators, which may affect the precision of our evaluation. Future research can further improve the indicator system through more extensive data collection and integration to enhance the accuracy and reliability of the assessment results. Additionally, in analyzing the driving factors of CCD, our investigation of six key factors, grounded in existing literature, might not have fully captured all relevant influences, considering the extensive range of factors impacting the CCD between these two systems. Future research should further expand the scope of drivers to include not only traditional factors such as economic, environmental, and social aspects, but also more dynamic and non-linear factors such as policy changes, technological advances, and cultural influences. Finally, in examining the convergence of CCD, this paper employs a Sys-GMM model, which effectively addresses the endogeneity issue by incorporating lagged terms of explanatory variables and differential lagged terms as instrumental variables. However, it still faces challenges posed by bidirectional causality, leading to potential bias in estimation results. Future research should explore alternative methodologies to minimize the impact of endogeneity problems as much as possible.

## Conclusions and recommendations

### Conclusions

Utilizing data from 56 cities in the YRB from 2003 to 2020, this study constructs assessment systems for LUEE and UER. The super-efficiency SBM model, entropy method, and the CCD model were employed to measure the two systems and the temporal and spatial evolution characteristics of their CCD. Additionally, Kernel density estimation and the convergence model are utilized to reveal the dynamic evolution trends and temporal convergence of the CCD between LUEE and UER, while the key driving factors are analyzed using the Geodetector method. In summary, the following conclusions are drawn:

(1) After dividing the YRB into three sub-regions, it is found that the lower reaches exhibit the highest levels of LUEE, UER, and CCD. Additionally, cities with high levels of CCD are primarily concentrated in the lower reaches and in some key cities.

(2) The Kernel density curve in the lower reaches demonstrates a significant “double peak” pattern, suggesting a polarized distribution of CCD, while the overall basin and its middle and upper reaches mainly show a single peak, without significant polarization.

(3) CCD in the overall YRB and its sub-regions exhibits *σ*-convergence, while the *β*-convergence coefficients are significantly negative. Additionally, the conditional *β*-convergence speed is faster than the absolute* β*-convergence speed.

(4) Regarding the effects of independent factors, the core driving force of CCD is the government fiscal budget expenditure. In terms of two-factor interactions, the interaction between industrial structure upgrading and the government fiscal budget expenditure is the most significant in influencing the CCD between LUEE and UER.

### Recommendations

To promote the coordinated and sustainable development of the YRB, we propose the following recommendations:

(1) Strengthen the radiation-driven role and promote regional coordinated development. Considering the high level of CCD in LUEE and UER in the lower reaches of the YRB and key cities, their radiation-driven role should be continuously leveraged. Utilizing their advantages in resources, technology, and policies can enhance land resource utilization efficiency and environmental management in neighboring cities. This will further promote the coordinated development of economic activities and ecological protection in the overall YRB and its sub-regions, thereby narrowing the regional development gap.

(2) Implement a tiered policy to promote the development of heterogeneous advantages. Given the phenomenon of polarization in the YRB, a tiered policy should be formulated to meet the needs of the different stages of development in the YRB. High-level cities should be encouraged to continue leveraging their strengths, enhance their innovative capacity, and maintain or improve their competitiveness, while also focusing on ecological protection and sustainable urban development. For low-level cities, the government should increase support with more policy, financial, and technical assistance to help improve their infrastructure, optimize industrial structures, and enhance LUEE and UER.

(3) Follow the principle of convergence to optimize regional development policies. We should formulate implementation policies based on the convergence trends and development levels of each region. It is crucial to fully consider the differences in regional resource endowments, adhere to the principle of “adapting to local conditions, applying policies according to different areas, and promoting synergistic development,” and pay attention to coordinating the convergence speed and development gap between LUEE and UER in each region of the YRB.

(4) Increase investment in environmental protection and promote the green transformation of industry. Given that government fiscal budget expenditure is crucial for enhancing the coordinated development of LUEE and UER, it is essential to continue increasing funding for ecological protection and land planning. Additionally, the need for upgrading the industrial structure should be fully considered, promoting the transformation of industries toward higher-end, environmentally friendly directions. At the same time, a comprehensive evaluation and monitoring mechanism should be established to regularly assess LUEE and UER, ensuring the effective implementation of policies.

## Data Availability

The datasets used and/or analysed during the current study available from the corresponding author on reasonable request.
